# Genome-wide analysis of functional sirtuin chromatin targets in yeast

**DOI:** 10.1186/gb-2013-14-5-r48

**Published:** 2013-05-27

**Authors:** Mingguang Li, Veena Valsakumar, Kunal Poorey, Stefan Bekiranov, Jeffrey S Smith

**Affiliations:** 1 Department of Biochemistry and Molecular Genetics, University of Virginia, School of Medicine, 1340 Jefferson Park Ave, Charlottesville, VA 22908, USA

## Abstract

**Background:**

The sirtuins are a conserved family of NAD^+^-dependent histone/protein deacetylases that regulate numerous cellular processes, including heterochromatin formation and transcription. Multiple sirtuins are encoded by each eukaryotic genome, raising the possibility of cooperativity or functional overlap. The scope and variety of chromatin binding sites of the sirtuins in any specific organism remain unclear.

**Results:**

Here we utilize the ChIP-seq technique to identify and functionally characterize the genome-wide targets of the sirtuins, Sir2, Hst1 to Hst4, and the DNA binding partner of Hst1, Sum 1, in *Saccharomyces cerevisiae*. Unexpectedly, Sir2, Hst1 and Sum1, but not the other sirtuins, exhibit co-enrichment at several classes of chromatin targets. These include telomeric repeat clusters, tRNA genes, and surprisingly, the open reading frames (ORFs) of multiple highly expressed RNA polymerase II-transcribed genes that function in processes such as fermentation, glycolysis, and translation. Repression of these target genes during the diauxic shift is specifically dependent on Sir2/Hst1/Sum1 binding to the ORF and sufficiently high intracellular NAD^+ ^concentrations. Sir2 recruitment to the ORFs is independent of the canonical SIR complex and surprisingly requires Sum1. The shared Sir2/Hst1/Sum1 targets also significantly overlap with condensin and cohesin binding sites, where Sir2, Hst1, and Sum1 were found to be important for condensin and cohesin deposition, suggesting a possible mechanistic link between metabolism and chromatin architecture during the diauxic shift.

**Conclusions:**

This study demonstrates the existence of overlap in sirtuin function, and advances our understanding of conserved sirtuin-regulated functions, including the regulation of glycolytic gene expression and condensin loading.

## Background

The sirtuins are a highly conserved family of NAD^+^-dependent protein deacetylases that regulate a wide range of cellular processes impacted during aging and in age-associated diseases such as type 2 diabetes and cancer (reviewed in [1]). They utilize NAD^+ ^as a co-substrate during the deacetylation reaction, such that one molecule of NAD^+ ^is converted into nicotinamide and 2'O-acetyl ADP-ribose for every lysine that is deacetylated [2,3]. As a result, sufficiently high NAD^+ ^concentrations are required to properly regulate cellular processes in which the sirtuins participate [4,5]. This link to NAD^+ ^gives sirtuins an inherent ability to 'sense' the intracellular energy status, and regulate target proteins via lysine deacetylation.

The genomes of eukaryotic organisms usually encode multiple sirtuin proteins. For example, there are seven sirtuins in mammals known as SIRT1 through SIRT7 [6], while the budding yeast *Saccharomyces cerevisiae *encodes five, known as Sir2, and Hst1 through Hst4 (homologs of Sir two) [7]. Sir2 is the founding family member [7], and was initially characterized as a factor required for transcriptional silencing at the *HML *and *HMR *silent mating-type loci, telomeres, and the ribosomal DNA (rDNA) tandem array, each of which have characteristics of heterochromatin in more complex eukaryotes (reviewed in [8]). Eventually, Sir2 was found to be a histone deacetylase [9,10], a seminal discovery that instantly provided a mechanistic role for Sir2 in the formation of heterochromatin. Equally exciting were the implications for aging because Sir2 was also characterized as a limiting factor for replicative life span [11], which is defined as the number of times a yeast mother cell divides before senescing. Deletion of *SIR2 *shortens replicative life span, while increased *SIR2 *gene dosage extends both mean and maximum replicative life span [11]. Similarly, SIRT6 knock out mice prematurely age [12], and male SIRT6 transgenics are long-lived [13], suggesting that longevity and/or health span support by sirtuins could be one of their conserved features.

Many histone-modifying enzymes are catalytic subunits of large multi-protein complexes, and the nuclear sirtuins appear to follow this trend. At the *HM *loci and telomeres, yeast Sir2 is associated with the Sir3 and Sir4 proteins in a complex known as SIR [14-16]. Deleting either of the SIR complex subunits results in a loss of transcriptional silencing [17,18]. At the rDNA locus, Sir2 associates with Net1 and Cdc14 in the nucleolar silencing complex known as RENT, which silences RNA polymerase II transcription from the intergenic spacers [19,20]. The Sir2 paralog, Hst1, forms a complex with Sum1 and Rfm1 (the Sum1 complex), which represses specific genes through localized histone deacetylation at promoters [21-23]. SIRT1 is considered the mammalian Sir2 ortholog, and like Sir2, is a histone deacetylase that can function in heterochromatin formation [24]. SIRT1 also regulates the expression of numerous genes and deacetylates numerous non-histone protein targets, but it has only recently been considered part of a larger multi-protein co-repressor complex [25]. Thus, sirtuins are generally recruited to multiple targets (chromatin or non-histone) through interactions with partner proteins that provide an added level of binding specificity. Given such diversity, complete inventories of conserved sirtuin targets, both chromatin and non-chromatin, are needed in order to understand how these factors are truly integrated with various cellular processes via NAD^+^.

In this study we have focused on the identification and characterization of novel chromatin targets of sirtuins in yeast, with the goal of uncovering additional cellular pathways that are impacted by alterations in cellular NAD^+^, and determining the extent of functional overlap between the various sirtuins. Conserved targets of this nature are also more likely to be mediators of longevity. We have utilized chromatin immunoprecipitation (ChIP), followed by next generation DNA sequencing (ChIP-seq), to obtain high-resolution chromatin association maps for each yeast sirtuin. Work presented in this study functionally characterizes the overlapping roles for Sir2, Hst1, and Sum1 in telomere maintenance, as well as NAD^+^-dependent repression of specific genes that are downregulated during the diauxic shift, a time in which yeast shift their metabolism from aerobic fermentation to respiration. These include genes involved in glycolysis, fermentation, and translation. Sir2, Hst1, and Sum1 were also required for the efficient recruitment of condensin and cohesin onto multiple shared target sites, including tRNAs and other genes downregulated during the diauxic shift.

## Results

From an earlier expression profiling study, we identified thiamine biosynthesis genes as being upregulated when yeast cells were treated with the sirtuin inhibitor nicotinamide, or when intracellular NAD^+ ^levels were reduced by deleting the NAD^+ ^salvage pathway gene *NPT1 *[26]. Hundreds of other genes were also upregulated, including known Hst1/Sum1 targets such as middle sporulation and NAD^+ ^biosynthesis genes. These results raised the question of whether each sirtuin specifically regulates a distinct set of gene targets? We therefore set out to obtain a genome-wide picture of binding sites for each sirtuin using ChIP-seq. The sirtuins were carboxy-terminally tagged either with 13 copies of the Myc epitope (Sir2, Hst1, and Hst2) or one copy of the TAP-tag (Hst3 and Hst4). Sum1 was also tagged with 13xMyc to test if it consistently tracked with its binding partner Hst1. Cells growing exponentially in rich YPD medium were then subjected to ChIP with anti-Myc or anti-TAP antibodies, and the recovered DNA sequenced. Certain genomic regions were previously shown to be over- or under-represented when genomic DNA was sequenced by this method [27]. Therefore, to control for representation in the ChIP libraries, crosslinked DNA that went into the immunoprecipitation (IP) reactions (the input) was also sequenced.

### Sirtuin binding at the silent mating-type loci and the rDNA tandem array

**Figure 1:**
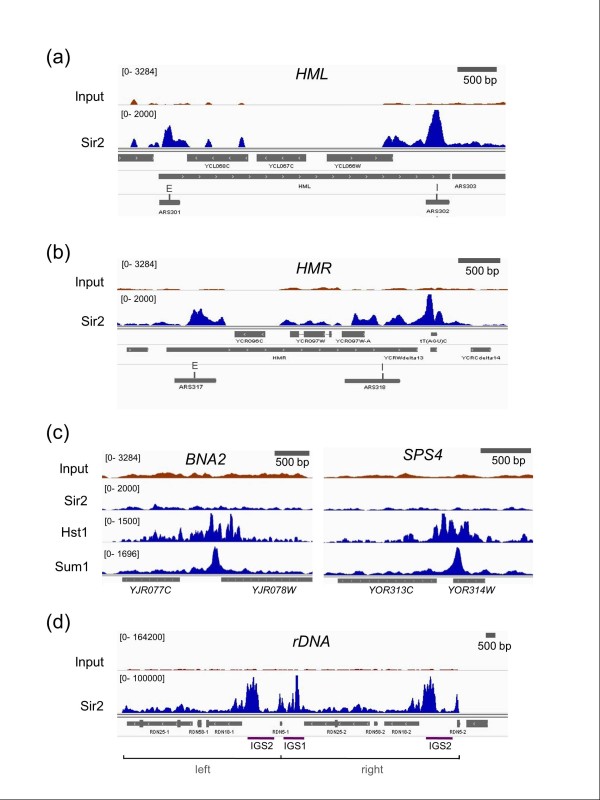
**High resolution ChIP-seq mapping at known Sir2, Hst1, and Sum1 targets**. **(a) **Sir2 enrichment at the *HML*. Locations of the E and I silencers are indicated. **(b) **Sir2 enrichment at the *HMR*. The right boundary element tRNA^Thr ^gene is indicated as tT(AGU)C. Locations of the E and I silencers are indicated. **(c) **Sir2, Hst1, and Sum1 enrichment at the *BNA2 *and *SPS4 *promoters. **(d) **Sir2 enrichment at the rDNA repeats on chromosome XII. The left-most and right-most repeats annotated in the SGD genome assembly are displayed, but the read counts in brackets are a compilation from all approximately 175 repeats. The 5S genes within the intergenic spacers are indicated as *RDN5-1 *and *RDN5-2*, and define the right end of each repeat.

We first analyzed Sir2 at the silent mating-type loci *HML *and *HMR *to confirm the ChIP-seq procedure was effective. Sir2 was clearly enriched at the *HML-E *and *-I *silencers as expected (Figure 1a). Sequence reads derived from repetitive regions are discarded by the mapping program, so there is a lack of binding information for the α1 and α2 genes within *HML *because they are duplicated at *MATα*. However, Sir2 was still noticeably enriched between the two silencers (Figure 1a), which supported the results of an earlier study suggesting unidirectional spreading from each silencer [28]. At *HMR *we observed a strong peak of Sir2 at the *HMR-E *silencer that extended rightward across the body of *HMR *as expected, but there was no corresponding peak at the *HMR-I *silencer (Figure 1b). Instead, a very strong peak of Sir2 was located at an adjacent tRNA^Thr ^gene, which had already been well characterized as a silencing boundary element [29]. Earlier ChIP results showed that Sir2 does not associate with *HMR-I *when the *E *silencer is defective, suggesting that Sir2 is not independently recruited to the I silencer, but rather spreads all the way across *HMR *until it reaches the boundary [30]. Our data suggest an extension of this model in which the tRNA^Thr ^gene plays a dual role as a boundary element and as a protosilencer where Sir2 is independently recruited. A protosilencer is defined as a *cis*-acting sequence that cannot establish silencing on its own, but instead cooperates in establishment and maintenance of the silent chromatin [31]. Support for such a protosilencer model comes from a recent study where the tRNA^Thr ^boundary element was found to impose the cell cycle progression requirement for establishing silencing at *HMR *[32].

There was no visible enrichment of Hst2, Hst3 or Hst4 at the silencers or across *HML *or *HMR*, but surprisingly, Hst1 and Sum1 were both highly enriched and co-localized with Sir2 at the tRNA^Thr ^boundary element flanking *HMR *(Figure S1 in Additional file 1), suggesting they could also potentially be involved in the cell cycle progression requirement in silencing linked to this *cis*-acting element. Given the unexpected co-localization of Sir2, Hst1, and Sum1 at the tRNA^Thr ^gene, we next asked whether Sum1 and Hst1 were properly associating with several of their known targets, including the promoters of specific NAD^+ ^biosynthesis and middle sporulation genes [21,33]. As shown in Figure 1c, Sum1 was clearly bound to such promoters (*BNA2 *and *SPS4*) with a single strong peak and without any indication of spreading, consistent with previous low-resolution ChIP assays [34]. Hst1 was also highly enriched at the same sites, but its occupancy was extended a short distance (one or two nucleosomes) in both directions (Figure 1c). Importantly, Sir2 does not repress these genes [35], and we did not detect any binding signal in the ChIP-seq profiles (Figure 1c). Taken together, these results indicated that the ChIP-seq data were highly specific, and the co-association of Sir2, Hst1, and Sum1 at the tRNA^Thr ^gene and other genomic sites (see below) is likely meaningful.

Sir2-dependent transcriptional silencing also occurs at the rDNA tandem array [36,37], which consists of approximately 150 to 200 identical rDNA gene copies on the right arm of chromosome XII. Only the leftmost and rightmost repeats are annotated, so ChIP-seq reads that map to these two repeats are a compiled average for the entire array (Figure 1d). The mapping program parameters were adjusted in this case to allow for repeated sequences, so the read counts for Sir2 peaks in the rDNA approached 200,000, compared to approximately 2,000 for *HML *and *HMR*. Consistent with previous silencing and ChIP results [38,39], Sir2 strongly associated with the rDNA intergenic spacers (IGS1 and IGS2), and its extended enrichment to the left of IGS2 occurs in the same direction as Pol I transcription (Figure 1d). The strongest Sir2 peak in IGS1 corresponded exactly to a replication fork block site (chromosome XII coordinates: 460530-460570), where Fob1 recruits the RENT and cohibin complexes [40]. While there may be some low-level association of Hst1, Sum1, Hst2, Hst3, and Hst4 across the rDNA, it was minimal compared to the overwhelming Sir2 signal (Figure S1 in Additional file), which is presumably derived from the RENT complex.

### Telomere length maintenance by Sir2, Hst1, and Sum1

**Figure 2:**
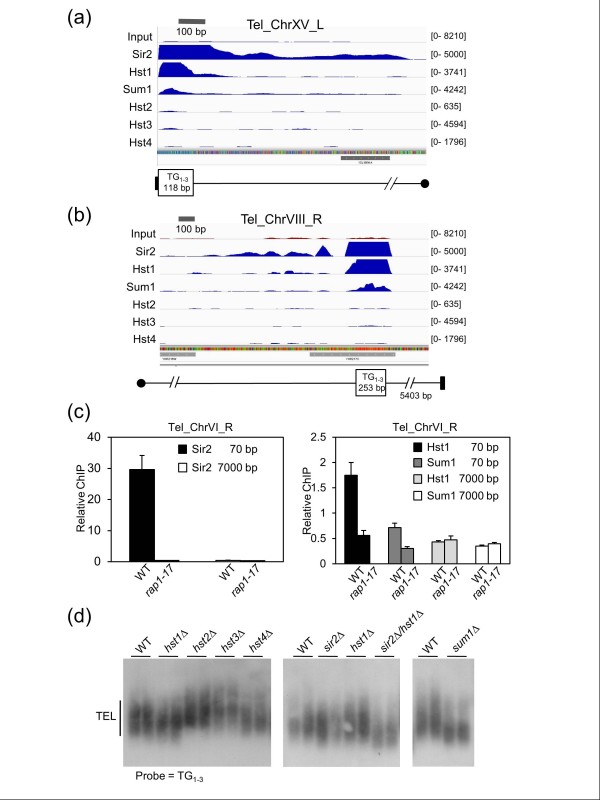
**Functional sirtuin enrichment at telomeric repeat clusters**. **(a) **Enrichment of Sir2, Hst1 and Sum1 at terminal TG_1-3 _repeats at the left telomere of chromosome XV (Tel_ChrXV_L). The black rectangle represents the end of the chromosome and the circle represents the centromere. **(b) **Enrichment of Sir2, Hst1 and Sum1 at an internal TG_1-3 _subtelomeric cluster on the right arm of chromosome VIII (Tel_ChrVIII_R). **(c) **Quantitative ChIP assays measuring the effect of a *rap1-17 *mutation on telomeric Sir2-myc enrichment (left panel) or Hst1-myc and Sum1-myc enrichment (right panel). The amplified region was either 70 bp or 7,000 bp away from the terminal TG_1-3 _cluster on the right arm of chromosome VI. The immunoprecipitated PCR signal is relative to the input chromatin PCR signal (Relative ChIP). In this experiment the untagged background signal was subtracted out. Error bars represent standard deviation. **(d) **Southern blot detection of telomere (TEL) lengths using a probe that hybridizes to poly (TG_1-3_) sequences. WT, wild type.

The third known type of silencing in yeast occurs at telomeres. The SIR complex is recruited to telomeres through physical interaction with the Rap1 protein, which directly binds to telomeric TG_1-3 _repeats [41,42]. As with the *HM *loci, sequence read coverage of many telomeric regions was sparse. When there was sufficient coverage, Sir2 association with the terminal TG_1-3 _repeats was observed (Figure 2a), but its relative distribution among different telomeres was quite variable (Table S1 in Additional file 1). Surprisingly, strong peaks of Hst1 and Sum1 binding were observed directly overlapping with Sir2 at the TG_1-3 _repeats, regardless of whether they were terminal or internal clusters (Figure 2a,b; Table S1 in Additional file 1). From binding sites identified using BayesPeak [43], we calculated the enrichment of Hst1, Sum1, or Sir2 for telomeric repeats was at least 30-fold greater than expected by chance (Table S2 in Additional file 1). Sir2 enrichment at terminal and internal TG_1-3 _clusters typically extended inward up to approximately 1 kb, whereas Hst1 and Sum1 were more restricted to the actual repeats (Figure 2a,b).

To test whether Hst1 and Sum1 were recruited to telomeric repeats via Rap1, their enrichment was assayed by quantitative ChIP in wild type (WT) and *rap1-17 *mutant strains [44]. The *rap1-17 *allele truncates the carboxy-terminal SIR interaction domain of Rap1, resulting in mis-localization of the SIR complex and severe silencing defects at the *HM *loci and telomeres [42,45]. The repetitive nature of telomeric TG_1-3 _sequences prevented direct real-time PCR amplification of these regions, so we instead assayed 70 bp adjacent to the cluster on the right arm of chromosome VI, or 7,000 bp away from the repeats as a control. As expected, strong telomeric enrichment of Sir2 at the 70 bp position was eliminated in the *rap-17 *mutant (Figure 2c, left panel). There was also a low level of Hst1 and Sum1 enrichment 70 bp from the TG repeats in the WT strain, confirming the poor spreading capability of these two proteins, relative to Sir2. Importantly, even this low level Hst1 and Sum1 occupancy was significantly reduced in the *rap1-17 *mutant (Figure 2c, right panel), suggesting that Rap1 significantly contributes to their recruitment. However, we cannot rule out the possibility that occupancy was indirectly reduced in the mutant because of the general defect in telomeric heterochromatin formation.

Given the precise association of Sir2, Hst1, and Sum1 with terminal TG repeats, we next tested for functional consequences of deleting the sirtuins or *SUM1 *on telomere length. As shown in Figure 2d, s*ir2Δ *or *hst1Δ *mutants had little effect on telomere lengths compared to WT strains, but the *sir2Δ hst1Δ *double mutant clearly had shorter telomeres on average, indicating significant redundancy between the two paralogs. The *sum1Δ *mutant also had short telomeres similar to the double mutant length. Compared to Sir2, Hst1, and Sum1, the occupancy of Hst2, Hst3, and Hst4 at the TG_1-3 _repeats was absent or extremely low (Figures 2a,b), suggesting the slightly longer telomeres in *hst2Δ *and *hst3Δ *mutants were likely an indirect effect (Figure 2d). Because Sir2, Hst1, and Sum1 appeared to have more specific co-enriched binding sites and functional redundancy than initially anticipated, we focused subsequent analysis on these three proteins.

### Sir2, Hst1, and Sum1 associate with and regulate highly transcribed genes that are downregulated during the diauxic shift

**Figure 3:**
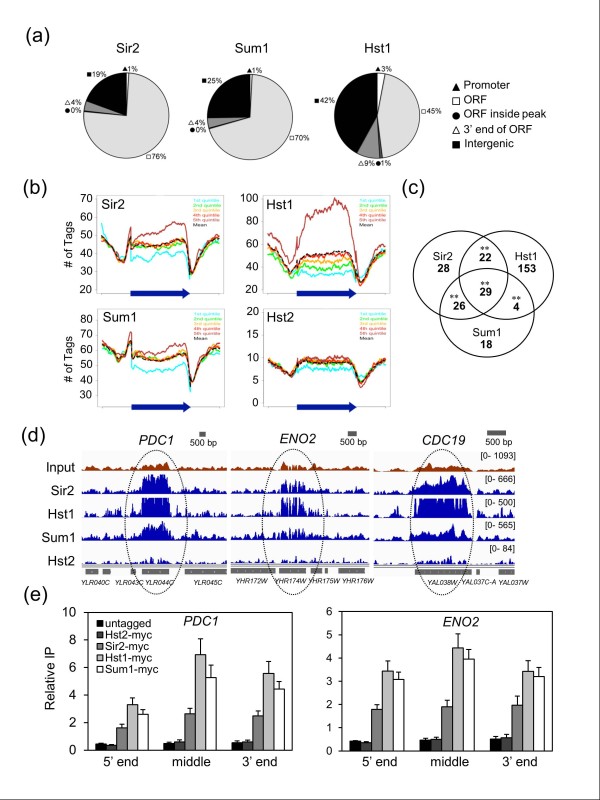
**Summary of Sir2, Hst1, and Sum1 binding across the yeast genome**. **(a) **Gene-based distribution of Sir2, Hst1, and Sum1 binding sites identified with BayesPeak. Filled circles represent relatively small ORFs that were completely embedded within a peak. **(b) **Composite plots showing that Sir2, Hst1, and Sum1, but not Hst2, are enriched at the most highly expressed genes. The sequence of each annotated ORF in the genome was normalized to 1,000 bins, with another 500 bins upstream and 500 downstream, into which the number of sequencing reads (tags) were distributed. The expression level of each ORF was divided into five quintiles, with the 5th quintile the highest. **(c) **Venn diagram showing the overlap between genes with Sir2, Hst1 or Sum1 are enriched across >60% of the ORF. **P-value <0.005. **(d) **Screenshots of Sir2, Hst1, Sum1, and Hst2 enrichment across the *PDC1*, *ENO2*, and *CDC19 *genes. Hst1 acts as a negative control similar to the Input sequence. **(e) **ChIP assay confirming the enrichment of Sir2, Hst1 and Sum1 across the ORFs of *PDC1 *and *ENO2*. Relative IP indicates the ratio of IP PCR signal to the input chromatin PCR signal for each sample. Error bars represent standard deviation.

Comparisons of BayesPeak-identified Sir2, Hst1 or Sum1 enrichment peaks to gene annotations revealed that the largest percentage of binding sites for each protein was surprisingly found within ORFs (Figure 3a). To determine if there was any relationship between ORF enrichment and expression levels for the associated genes, composite plots were generated for Sir2, Hst1, Sum1, or Hst2 ChIP-Seq read numbers across all yeast genes, with the expression levels divided into five quintiles (Figure 3b). Sir2, Hst1, and Sum1 enrichment, but not Hst2, was strongest toward the 3' end of ORFs with the highest expression levels (5th quintile). There was also significant overlap between the ORFs bound by each factor (Figure 3c), which was easily visualized in the genome browser for highly expressed genes such as *PDC1*, *ENO2*, and *CDC19 *(Figure 3d). Traditional ChIP assays were performed with several target genes to confirm the ORF enrichment was not a sequencing artifact (Figure 3e; Figure S2a in Additional file 1). Hst2 did not have this pattern of binding in the composite plots or in the genome browser, and was not ORF-enriched in standard ChIP assays compared to an untagged control (Figure 3e), consistent with its reported cytoplasmic localization pattern [46].

**Figure 4:**
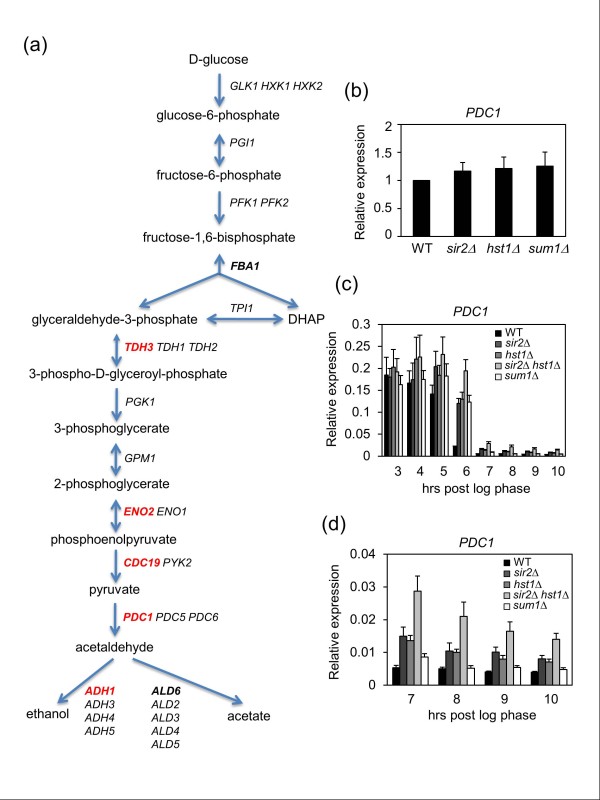
**Repression of *PDC1 *by Sir2 and Hst1 during the diauxic shift**. **(a) **Overview of the glycolysis/fermentation pathway in yeast. Red lettering indicates genes bound by Sir2, Hst1 and Sum1 across the ORF. Bold lettering indicates the genes only bound by Sir2. **(b) **Quantitative RT-PCR analysis of *PDC1 *mRNA levels in log phase WT and mutant strains. Expression level in the WT strain was normalized to 1.0. **(c) ***PDC1 *expression level in WT and deletion strains when progressing through the diauxic shift. **(d) **Enlarged image of (c) showing the later time points. Error bars represent standard deviation.

Many of the most highly expressed genes during log phase tend to be strongly repressed when cells enter the diauxic shift. We used publicly available expression profiling data [47], to ask whether genes with Sir2, Hst1, or Sum1 binding on their ORFs were differentially regulated during the diauxic shift (Figure S2b in Additional file 1). For each individual factor there was a trend for association with downregulated genes, which reached strong statistical significance for Hst1. Coupled with this trend, there was also a highly significant depletion for upregulated genes (Figure S2b in Additional file 1,). Furthermore, genes with overlapping Sir2, Hst1, and Sum1 ORF binding were enriched for Gene Ontology terms related to glycolysis, glucose fermentation, translation, and cell wall biosynthesis (Table S3 in Additional file 1), all processes that are highly active during log phase and then repressed during the diauxic shift [48]. Five of the ORF-targeted genes encode enzymes involved in glucose fermentation (Figure 4a, highlighted in red), so we hypothesized that expression of such genes would be dysregulated when *SIR2*, *HST1*, or *SUM1 *were deleted. No significant differences in *PDC1 *or *ENO2 *expression were observed in the deletion mutants during log phase (Figure 4b; Figure S3a in Additional file 1), but they were not properly repressed when entering the diauxic shift (Figure 4c; Figure S3b in Additional file 1). *PDC1 *and *ENO2 *in the WT strain were strongly repressed within 6 hours after log phase, while repression in the mutants was significantly delayed. This was especially true in the *sir2Δ hst1Δ *double mutant, which was unable to fully repress *PDC1 *even 10 hours after log phase (Figure 4d), suggesting some redundancy between Sir2 and Hst1, as was also observed in telomere length regulation (Figure 2d).

**Figure 5:**
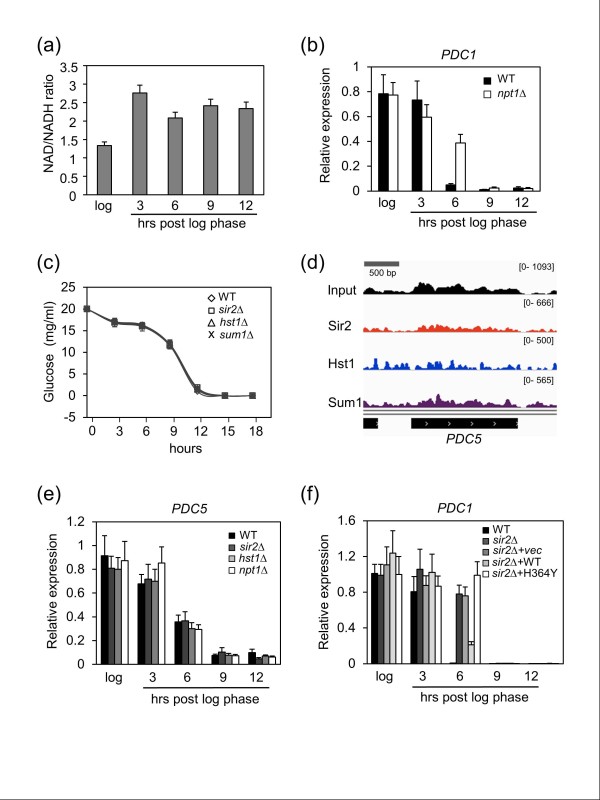
**Effects of intracellular NAD^+ ^and Sir2 activity on the expression of *PDC1 *during the diauxic shift**. **(a) **Relative NAD^+^/NADH ratios when WT strain was grown in YPD at log or post log phase. **(b) ***PDC1 *expression in WT and *npt1Δ *strains growing in YPD medium. **(c) **Extracellular glucose concentrations as WT, *sir2Δ*, *hst1Δ*, and *sum1Δ *strains enter the diauxic shift. Cells were grown in YPD. **(d) **Chromosome mapping showing no significant binding of Sir2, Hst1 and Sum1 across the *PDC5 *ORF. **(e) ***PDC5 *expression in WT and mutant strains during the diauxic shift. **(f) **Effect of a catalytically inactive Sir2-H364Y mutant protein on *PDC1 *expression during the diauxic shift. Error bars represent standard deviation.

During the diauxic shift, cellular metabolism switches from fermentation to respiration, which causes an increase in the NAD^+^/NADH ratio. We observed this increased ratio in the WT strain within 3 hours post log phase (Figure 5a). Since the higher NAD^+^/NADH ratio induced by calorie restriction has been reported to promote Sir2 function [49], we next tested whether simply reducing the intracellular NAD^+ ^concentration by deleting *NPT1 *would also delay *PDC1 *repression, which it did (Figure 5b). Similar results were observed for *ENO2 *and *CDC19 *(Figure S3c in Additional file 1). The time course of glucose consumption from the YPD medium was almost identical between the WT strain and the *sir2Δ*, *hst1Δ*, or *sum1Δ *mutants (Figure 5c), but it was still formally possible that the mutants were not properly repressing *PDC1 *simply because their entry into the diauxic shift was delayed. If this were the case, then other genes down-regulated at the diauxic shift whose ORFs were not bound by Sir2, Hst1, or Sum1 should also show a delay in repression in the mutant strains. *PDC5 *encodes a minor isoform of pyruvate decarboxylase (Figure 4a), but does not have Sir2, Hst1, or Sum1 enriched on the ORF (Figure 5d). Importantly, *PDC5 *repression at the diauxic shift occurred normally in the *sir2Δ*, *hst1Δ*, or *npt1Δ *mutants (Figure 5e). To make sure this was not a peculiarity of *PDC5*, we tested three additional genes from this class (*CLB1*, *RIF1*, and *RPB3*) in the *sir2Δ *and *npt1Δ *mutants, and again did not observe any defect in repression across the time course (Figure S4 in Additional file 1). These results suggest a model where Sir2 and Hst1 are associated with specific targeted ORFs in a poised state during log phase, and then become functional for transcriptional repression at onset of the diauxic shift, perhaps aided by the increased NAD^+^/NADH ratio. This would result in the observed early and rapid gene repression, probably before the overall reduction in the general transcription machinery occurs.

We initially hypothesized that Sir2 and Hst1 function in repression of their ORF-targeted genes by deacetylating histones across the ORF when cells enter the diauxic shift. However, using quantitative ChIP, there was no consistent increase in various histone H3 or H4 acetylation marks on the *PDC1 *or *ENO2 *genes in *sir2Δ*, *hst1Δ*, *sum1Δ*, or *npt1Δ *mutants during log phase or the diauxic shift (data not shown). Thinking that there could be some redundancy involved, *sir2Δ sum1Δ *and *sir2Δ hst1Δ *double mutants were also tested, but H3 or H4 acetylation was still not elevated compared to WT (Figure S5 in Additional file 1). Even so, the requirement for high NAD^+ ^concentration in Figure 5b strongly suggested that Sir2 and/or Hst1 catalytic activity was required for the repression. The effect of an H364Y mutation in *SIR2 *that eliminates deacetylase activity was tested and found to block *PDC1 *repression at onset of the diauxic shift (Figure 5f), similar to the *sir2 *deletion. Therefore, the mechanism of repression remains unknown, but current evidence points toward either non-traditional lysine modifications or non-histone deacetylation targets such as RNA polymerase or elongation factors being important (see Discussion).

### Evidence for novel Sir2 and Hst1 complexes on targeted ORFs

**Figure 6:**
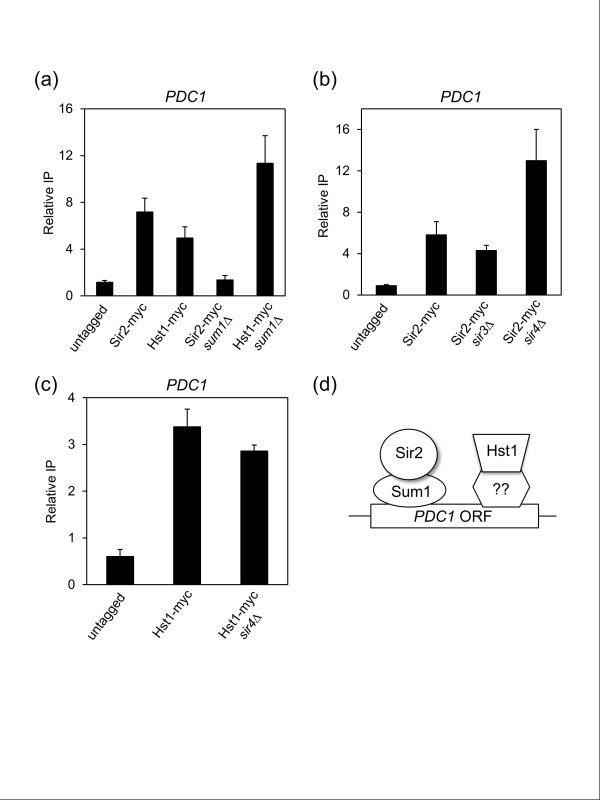
**Sum1-dependent recruitment of Sir2 to the *PDC1 *ORF**. **(a) **ChIP assay showing Sir2-myc and Hst1-myc enrichment at the *PDC1 *ORF in WT and *sum1Δ *strains. **(b) **ChIP assay showing Sir2-myc enrichment in WT, *sir3Δ*, and *sir4Δ *strains. **(c) **ChIP assay showing Hst1-myc enrichment in WT and *sir4Δ *strains. The relative IP in each panel indicates the IP PCR signal divided by the input chromatin PCR signal. (d) Model of independent recruitment of Sir2 and Sum1 to the *PDC1 *ORF. In this model, Sir2 is recruited via Sum1, while Hst1 is recruited by an unidentified factor. Error bars represent standard deviation.

The apparent inactivity of Sir2 and Hst1 on histones at the targeted ORFs suggested they could be functioning independently of their canonical HDAC complexes at these sites. To check this possibility, we first tested whether Sir2 or Hst1 enrichment on the *PDC1 *ORF was altered in a *sum1Δ *mutant. Sir2 enrichment was surprisingly lost, while Hst1 was increased (Figure 6a), strongly suggesting that Sum1 is involved in recruiting Sir2, but not Hst1. We next tested whether Sir2 or Hst1 enrichment was altered in *sir3Δ *or *sir4Δ *mutants. As shown in Figure 6b, Sir2 enrichment was maintained in both mutants, and actually increased in the absence of *SIR4*, reminiscent of the increased nucleolar Sir2 localization and rDNA silencing in a *sir4Δ *mutant caused by redistribution of the telomeric Sir2 pool [50,51]. Similarly, increased Hst1 enrichment in the *sum1Δ *mutant could be due to release of Hst1 from its traditional promoter targets. Since Sum1 appeared to be recruiting Sir2, we thought Hst1 may behave oppositely as well, and be recruited via interactions with Sir4. However, Hst1 enrichment at *PDC1 *was unaffected in the *sir4Δ *mutant (Figure 6c). Taken together, these ChIP results suggest a model whereby Sir2 is recruited to its ORF targets via Sum1, while Hst1 is recruited through an unknown bridging factor (Figure 6d). It is important to note that independent mechanisms of Sir2 and Hst1 recruitment are consistent with their observed functional redundancy in gene repression during the diauxic shift (Figure 4).

### Sir2, Hst1, and Sum1 promote condensin and cohesin recruitment to multiple chromatin loci, including tRNA and other diauxic shift-repressed genes

**Figure 7:**
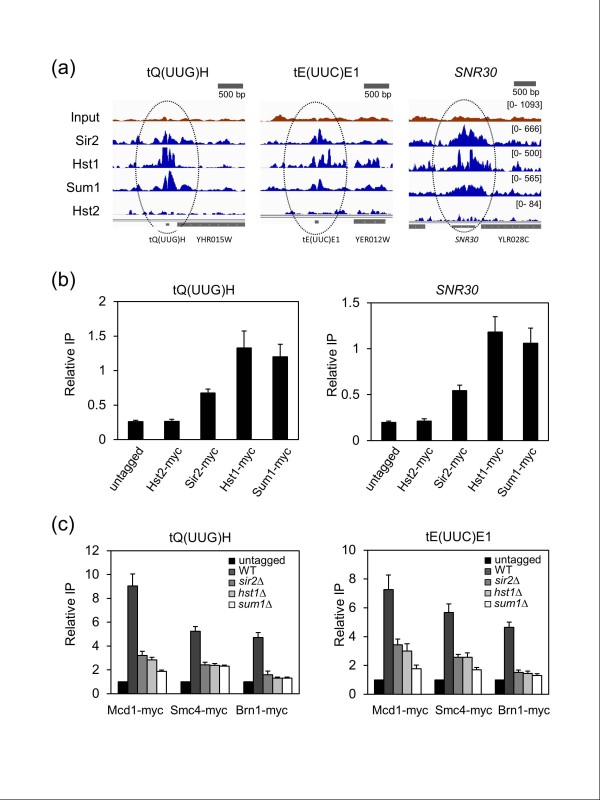
**Co-localization of Sir2, Hst1, and Sum1 with cohesin and condensin at Pol III-transcribed genes**. **(a) **IGV screenshots showing enrichment of Sir2, Hst1, and Sum1 with three Pol III-transcribed genes. Hst2 behaves as a negative control. **(b) **ChIP assay confirming the enrichment of Sir2, Hst1 and Sum1. **(c) **The decreased binding of a cohesin subunit (Mcd1) and two condensin subunits (Smc4 and Brn1) to the tRNA genes when *SIR2*, *HST1 *or *SUM1 *are deleted. Cells were grown in YPD to the log phase. The relative IP in each panel indicates the IP PCR signal divided by the input chromatin PCR signal, for normalization. Error bars represent standard deviation.

We earlier noted significant enrichment of Sir2, Hst1, and Sum1 at the tRNA^thr ^boundary element adjacent to *HMR-I *(Figure 1b). Subsequent binding site identification on Pol III-transcribed genes using BayesPeak revealed fold enrichment values of between 110- and 169-fold over random expectation (Table S4 in Additional file 1). There was also significant overlap between the Sir2, Hst1, and Sum1 associated genes (Figure S6 in Additional file 1). Example Integrative Genomics Viewer (IGV) screenshots for tQ(UUG)H, tE(UUC)E1, and *SNR30 *binding are shown in Figure 7a, revealing that these small genes are often the center of a broader peak for all three proteins. Binding to tQ(UUG)H and *SNR30 *was confirmed by standard ChIP assays (Figure 7b).

tRNA genes and the associated TFIIIC binding sites are well established as being chromatin insulator and boundary elements in yeast and human cells [29,52]. In yeast, the condensin and cohesin complexes have previously been demonstrated through ChIP-chip experiments to globally associate with tRNA genes [53,54]. We were curious if there was significant overlap of Sir2, Hst1, or Sum1 sites with the published cohesin or condensin binding sites. Indeed, the peaks for all three tagged proteins showed significant overlap with condensin peaks (approximately 140- to 200-fold over random) and cohesin peaks (approximately 24- to 29-fold over random) (Table S5 in Additional file 1). Sir2 and Hst1 were previously shown to function in cohesin recruitment to the silenced rDNA and *HMR *loci in yeast [55,56], but such a relationship with condensin had not been reported. To test whether Sir2, Hst1, and Sum1 are involved in the loading of condensin and/or cohesin onto tRNA genes, the condensin subunits Brn1 and Smc4, and the cohesin subunit Mcd1 were carboxy-terminally tagged with the 13xMyc epitope in WT, *sir2Δ*, *hst1Δ*, and *sum1Δ *strains. Steady state protein expression was equivalent in all the strains, as measured by western blotting with the 9E10 α-Myc antibody (Figure S7a in Additional file 1). ChIP assays for the tagged subunits revealed a strong dependence for Sir2, Hst1, and Sum1 in loading both condensin and cohesin onto tQ(UUG)H and tE(UUC)E1 (Figure 7c).

**Figure 8:**
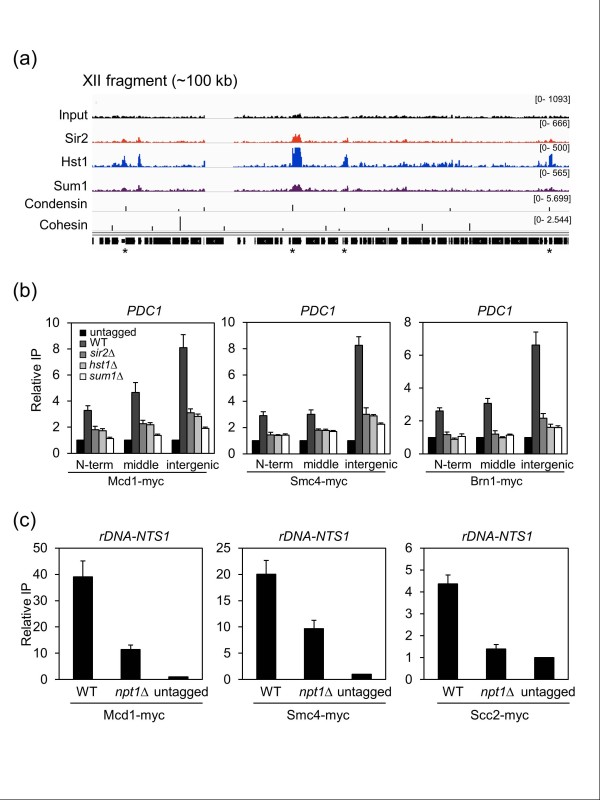
**Enrichment of cohesin and condensin at the *PDC1 *gene and rDNA is dependent on *SIR2*, *HST1*, *SUM1 *and *NPT1***. **(a) **Zoomed out view of a 100 kb chromosome XII region with four ORFs with Sir2, Hst1, and Sum1 enrichment that coincide with a condensin peak (asterisks). **(b) **The reduced binding of cohesin and condensin subunits to the *PDC1 *ORF when *SIR2*, *HST1 *or *SUM1 *was deleted. Cells were grown in YPD to log phase. **(c) **Reduced association of cohesin (Mcd1) and condensin (Smc4) subunits to the *rDNA *when *NPT1 *was deleted. Scc2 is a subunit of the Scc2/4 cohesin loading complex. The relative IP in each panel indicates the IP PCR signal divided by the input chromatin PCR signal, for normalization. Error bars represent standard deviation.

The links between Sir2, Hst1, and Sum1 with condensin and cohesin were not limited to Pol III-transcribed genes. Pol II-transcribed genes such as *PDC1 *with Sir2, Hst1, and Sum1 coating the ORF often had a condensin peak located near the 3' end of the gene (Figure 8a). This was intriguing given the trend for Sir2, Hst1, and Sum1 association toward the 3' ends of genes (Figure 3b). Quantitative ChIP demonstrated that condensin (Brn1 and Smc4) and cohesin (Mcd1) were indeed highly enriched in the intergenic region 3' of *PDC1 *and *ENO2 *(Figure 8b). But interestingly, significant levels of association were also observed across the ORFs. Regardless of the location tested, or the level of enrichment, the *sir2Δ*, *hst1Δ*, and *sum1Δ *mutations impaired the condensin/cohesin recruitment (Figure 8b), which was also observed for targeted ribosomal protein genes (Figure S7b in Additional file 1). We were unable to detect direct physical interactions between Sir2 and tagged condensin subunits through co-IP assays (data not shown), suggesting the recruitment may not be mediated by a direct physical interaction, but perhaps related to Sir2 catalytic activity. Consistent with that idea, the *npt1Δ *mutant with reduced NAD^+ ^caused a partial defect in cohesin (Mcd1) or condensin (Smc4) subunit association at the rDNA (Figure 8c). Furthermore, loss of Npt1 reduced enrichment of a cohesin loading factor subunit (Scc2), which may also promote functional condensin association with chromosomes [53]. Since condensin and cohesin mediate long-range chromatin interactions, this suggests that sirtuins could potentially make upstream contributions to this type of chromatin organization. Potential implications are addressed in the discussion.

## Discussion

### Overlapping functions of Sir2 and Hst1 mediated by Sum1

One of the unexpected findings from this study was the large number of locations where Sir2, Hst1, and Sum1 were co-enriched. Sir2 and Hst1 are paralogs that have acquired differential functions, but are still similar enough to substitute for one another under specific circumstances [35]. For example, a dominant *SUM1-1 *mutation suppresses the *HM *silencing defect of strains deleted for *SIR *genes by directing Hst1 to the silencers [57,58]. *HST1 *overexpression can also suppress the *HM *silencing defects of a *sir2Δ *mutant [7], and Sir2 can partially substitute for Hst1 in an *hst1Δ *background to suppress middle sporulation genes during vegetative growth [35]. It should be noted that Sum1 has repressive activity at the *HMR-E *silencer and some meiosis genes that is independent of Hst1 [33,59]. Perhaps Sum1 interacts with Sir2 at such locations when Hst1 is missing, which could even be mediated by another adaptor protein, similar to the role that Rfm1 plays in facilitating Hst1 interactions with Sum1 [22]. Our unexpected finding that Sum1 is required for Sir2 enrichment at the *PDC1 *ORF supports this hypothesis, and reveals even more diversity in the mechanism of chromatin targeting for Sir2 than was anticipated. However, the Sir2 recruited to ORFs via Sum1 is likely a minor subset of the overall Sir2 population, as indicated by the relatively lower Sir2-myc ChIP-seq read counts at ORFs compared to telomeres, the rDNA, and *HM *loci. Furthermore, Sir2 interaction with Sum1 in co-IP experiments has only been observed when *HST1 *is deleted [35], implying that most Sir2 is not associated with Sum1. Increased Sir2 enrichment in the *sir4Δ *mutant is likely due to redistribution from telomeres, and suggests the targeted ORFs are in competition with telomeres and rDNA for limiting Sir2. The mechanism of Sum1 and Hst1 recruitment to specific ORFs remains unknown, and merits future investigation.

We were initially surprised to detect strong Hst1 and Sum1 binding to the telomeric TG_1-3 _repeats, but genome-wide Southern blotting screens for deletion mutants with altered telomere lengths independently identified *sum1Δ *as having short telomeres [60,61]. Our results confirm the role of Sum1 in telomere maintenance, but also implicate Sir2 and Hst1, which appear to compensate for each other when one or the other is deleted. It had been speculated that the effect of the Sum1 complex on telomere length was likely an indirect effect on expression of upstream regulators of telomere length [61]. However, the precise association of Sum1 and Hst1 with the TG repeats strongly suggests that the SIR and Sum1 complexes have more direct functions in protecting the telomere ends. Rap1 recruits the SIR complex to telomere repeats [42], and we have now shown that Hst1 and Sum1 telomeric recruitment also depends on Rap1. Interestingly, there is a positive genetic interaction (*P *= 1.7e-06) between *RAP1-damp *and *sum1Δ *alleles in the DRYGIN database [62], supporting the idea of a functional relationship.

### tRNAs, sirtuins, and repression of adjacent Pol II-transcribed genes

We previously reported that Hst1 and Sir2 both directly repress basal expression of the thiamine biosynthesis gene *THI4 *by binding to and deacetylating histone H4 at a region approximately 700 to 800 bp upstream of the transcription start site [26]. That region happens to overlap with the tG(UCC)G gene, which, like many other Pol III-transcribed genes in the dataset, is co-enriched for Sir2, Hst1, and Sum1 binding (Table S4 in Additional file 1, and data not shown). We now hypothesize the tG(UCC)G gene is a *cis*-acting repressive element in this context, and that other tRNA genes with Sir2, Hst1, and Sum1 co-binding may repress adjacent Pol II-transcribed genes under specific growth conditions. Not all tRNA genes showed strong Sir2/Hst1/Sum1 co-enrichment, so any repressive effect would be limited to a subset of tRNA genes, including those that are within sufficient distance to a susceptible promoter, like *THI4*. Consistent with this model, an earlier genomic analysis found that Pol II-transcribed genes with a tRNA gene located within approximately 400 bp of the promoter were expressed approximately 3.5-fold less than genes without an adjacent tRNA gene [63]. A more recent study also showed that tRNA genes are often associated with Sir3 and Sir4, and those with strong DNA replication pausing activity can enhance silencing when positioned adjacent to an *ADE2 *reporter gene flanked by the *HMR-E *and *-I *silencers [64].

The Engelke lab previously described a form of tRNA gene-mediated (TGM) silencing where the *SUP4 *or *SUP53 *tRNA genes repress expression of an adjacent *HIS3 *reporter gene [65]. This silencing is dependent on the clustering of tRNA genes at the nucleolar periphery, but does not appear related to *HM*, telomeric, or rDNA silencing, because deleting the *SIR *genes has no effect [54,66]. Instead, TGM silencing is dependent on the association of condensin and cohesin with tRNA genes [54,67]. It is therefore intriguing that deletion of *SIR2*, *HST1*, or *SUM1 *causes a reduction in condensin and cohesin association with targeted tRNA genes (Figure 7c), but *sir2Δ *does not affect TGM silencing when tested with the reporter gene system [66]. The enrichment of Sir2, Hst1, and Sum1 is relatively weak at *SUP4 *and *SUP53 *compared to many other tRNA genes (data not shown), which could partially explain why their absence has little impact on TGM silencing, which is typically measured in the context of a plasmid. Alternatively, there could be sufficient redundancy between Hst1, Sum1, and Sir2 in condensin and cohesin recruitment to tRNA genes.

### The diauxic shift, glycolysis and sirtuins

*Saccharomyces cerevisiae *is a facultative anaerobe that ferments glucose even in the presence of oxygen. During log phase, genes involved in glycolysis, fermentation and growth-related processes are highly expressed, whereas genes involved in the tricarboxylic acid cycle and mitochondrial respiration are repressed [48]. As glucose is depleted, the cells undergo a change in gene expression and metabolism called the diauxic shift, during which the cells switch from glycolysis to ethanol catabolism and mitochondrial respiration, and prepare for survival in stationary phase (G_0_). Gene upregulation during the diauxic shift is mediated by transcriptional activators such as Msn2/Msn4 and Gis1, which turn on specific genes in response to the reduction in nutrients [68]. Specific mechanisms of transcriptional repression during the shift are less well understood, and are generally believed to be caused by downregulation and inactivation of the polymerase machinery [68], rather than by specific repressors. The results from our study indicate that Sir2, Hst1, and Sum1 directly contribute to the repression of genes to which they are bound across the ORF. The repression does not appear to involve traditional H3 or H4 deacetylation like that observed at the silenced domains (Figure S5 in Additional file 1), although we cannot rule out other less studied acetylation sites or alternative lysine modifications being important [69]. One attractive model is that the polymerase machinery or transcription elongation factors associated with the ORFs during transcription are inactivated by direct deacetylation during the diauxic shift. While it remains unclear if such factors are acetylated in yeast, several conserved RNA polymerase and elongation factor subunits have been identified as acetylated proteins in human cells [70]. In such a model, deacetylation by Sir2 and Hst1 could be partially triggered by the higher NAD^+^/NADH ratio that occurs at the onset of the diauxic shift (Figure 5a), with additional nutritional signaling inputs also likely involved. An alternative, and not mutually exclusive, model is that a repressive higher-ordered chromatin structure is established at these ORFs during the diauxic shift via the Sir2- and Hst1-mediated recruitment of condensin and cohesin (see below).

Earlier ChIP-chip studies on Sir2, Sir3, or Sir4 binding sites identified several euchromatic targets, but either did not test whether such genes were regulated by the SIR complex, or were unable to detect any expression changes in the SIR mutants [71-73]. We also did not observe changes in target gene expression during log phase growth (Figure 4b), but instead uncovered the role in repression of targeted genes downregulated at the diauxic shift, including glycolytic genes. This regulation of glycolytic genes by Sir2 and Hst1 is reminiscent of Sirt6 regulation of glycolytic genes in the mouse [74], where Sirt6 functions as a co-repressor for Hif1α, a critical regulator of nutrient stress. Sirt6 knock out mice upregulate genes involved in glycolysis and glucose import, and have reduced mitochondrial respiration activity [74]. During exponential growth in glucose, yeast cells almost exclusively ferment, and it is only at the diauxic shift when they will normally begin to respire. In this sense, yeast cells are similar to tumor cells that obtain much of their energy from aerobic glycolysis (the Warburg effect). Like mouse Sirt6, yeast Sir2 and Hst1 appear to favor the normal shift to respiration by repressing glycolytic genes when needed. A key difference is that Sirt6 appears to deacetylate histone tails at promoters [74] to repress genes, while Sir2 and Hst1 associate with the ORFs and likely deacetylate non-histone proteins. Since the original submission of this manuscript, Sirt6 has also been found to localize on specific ORFs [75], making the parallel between yeast and mammals even more compelling.

Biochemically, Sirt6 is a histone H3-K9 deacetylase that is required for proper telomere function and maintenance [76]. More recently, male Sirt6-transgenic mice were shown to have significantly extended lifespans [13], which does not happen with other sirtuin transgenics, and is consistent with the effect of increased Sir2 expression in yeast replicative life span [11]. From a life span standpoint, Sir2 appears to be functionally more similar to Sirt6 than Sirt1, a position that is now further supported by our findings that Sir2 and Hst1 both regulate glycolytic gene expression and are required for proper telomere maintenance. It is therefore tempting to speculate that these non-traditional functions for Sir2 could be related to its role in promoting longevity.

### Sirtuins and condensin/cohesin

Sir2-dependent recruitment of cohesin to the *HM *loci and rDNA is well established [40,55,77]. Mutations in cohesin subunits also result in rDNA silencing and recombination suppression defects [55], as well as impair the silencing boundary function of tRNA genes [78,79]. Interestingly, the tRNA^Thr ^boundary element next to *HMR-I *was previously shown to be important for establishing cohesion across the *HMR *locus, but silencing of *HMR *in the absence of the tRNA gene was not sufficient for establishment [80]. The finding that Sir2 contributes to efficient recruitment of cohesin to tRNA genes helps explain this observation. Sum1 and Hst1 are required for efficient cohesin recruitment at their shared target locations such as tRNA genes (Figure 7c), and therefore likely contribute to sister chromatid cohesion at those locations. We also found that high NAD^+ ^levels are critical for cohesin deposition, suggesting that sirtuin activity is involved. However, a catalytically inactive Sir2 protein can establish cohesion when it is targeted as a fusion protein adjacent to *HMR *in place of the tRNA^Thr ^gene [56]. Perhaps Hst1 is providing a redundant deacetylation function in this context.

We were compelled to assay for Sir2-mediated recruitment of condensin because of the highly significant overlap of Sir2, Hst1, and Sum1 binding sites with condensin binding sites, especially tRNA genes [53,54]. The negative effects of deleting *SIR2*, *HST1*, or *SUM1 *on condensin association with tRNA genes, the rDNA, or specific ORF regions were almost identical to the effects on cohesin, suggesting that their recruitment to these locations may involve a common factor. One possibility is the Scc2-Scc4 complex, which loads the cohesin complex onto chromosomes [81], but has also been suggested to contribute to condensin loading onto tRNA genes [53]. Consistent with this idea, Scc2 enrichment at the rDNA was impaired in an *npt1Δ *mutant (Figure 8c). A recent Sirt1 study showed that it was involved in the binding of condensin during mitotic chromosome condensation [82], implying another conserved link between yeast and mammalian sirtuin functions. Taken together, the requirements for Sir2, Hst1, and elevated NAD^+ ^concentrations in cohesin and condensin deposition at tRNA genes and genes downregulated at the diauxic shift suggest they may have significant impact on long-range chromosomal architecture beyond their traditional silenced targets.

## Conclusions

The budding yeast *S. cerevisiae *was used as a model system in identifying functional chromatin binding targets for all five sirtuins in this organism. A significant amount of overlap was surprisingly observed between Sir2 and Hst1, which was not shared with Hst2, Hst3, or Hst4. Importantly, these overlapping binding sites were not the previously described functional targets of Sir2 (silenced domains) or Hst1 (promoters of specific genes), but were instead novel targets. Binding of Sir2 and Hst1 at each new target class was functional, including telomere length maintenance at telomeric repeat clusters, diauxic shift-specific repression of glycolytic and translation factor genes through the binding to ORFs, and promoting condensin and cohesin deposition at overlapping positions. Sirtuins have numerous non-histone targets, but the results from this study highlight the idea that the chromatin targets of this enzyme class are equally broad and more highly conserved between yeast and human than previously thought.

## Materials and methods

### Yeast strains and media

Yeast strains were grown in YPD medium containing 2% glucose as the carbon source. All yeast growth was performed at 30°C. The strains used in this study were derived from the GRF167/JB740 strain background used for Ty1 and rDNA silencing studies [37], with the exception being a *rap1-17 *strain set in the W303 background [44]. All strains are listed in Table S6 in Additional file 1. Deletion strains were created by deleting each ORF and replacing them with either *kanMX4 *or *natMX4*, using one-step PCR-mediated gene replacement, and then PCR confirmation. Myc tagged strains were produced by fusing 13 copies of the Myc epitope (EQKLISEEDL) at the end of each gene.

### ChIP-sequencing

Log-phase cultures (200 ml) in YPD were cross-linked with 1% formaldehyde for 20 minutes at 30°C. Cells were pelleted by centrifugation and washed two times with cold Tris-buffered saline. The cells, in 0.6 ml of FA-140 lysis buffer (50 mM HEPES, 140 mM NaCl, 1% Triton X-100, 1 mM EDTA, 0.1% SDS, 0.1 mM phenylmethylsulfonyl fluoride, 2 mM benzamidine, 1× protease inhibitor cocktail (Sigma; St. Louis, MO, USA) were lysed with glass beads in a Mini-BeadBeater (Biospec Products; Bartlesville, OK, USA). The cell lysate was drawn off the beads, sonicated for 60 cycles (30 s 'on' at high level and 30 s 'off' per cycle) in a Bioruptor (Diagenode; Denville, NJ, USA) and spun for 10 minutes at 16,000x g in a microcentrifuge. Equivalent amounts of lysate (2.5 mg protein) were incubated overnight at 4°C with 5 µg of anti-Myc antibody (9E10) and 20 µl of protein G magnetic beads (Millipore; Billerica, MA, USA). The immunoprecipitated chromatin was then recovered and the DNA purified using a Magna ChIP™ G Chromatin Immunoprecipitation Kit (Millipore). The ChIP-sequencing libraries were made using an Illumina ChIP-Seq DNA Sample Prep Kit (catalogue number IP-102-1001), starting with 0.5 µg of DNA isolated from the immunoprecipitation step. The libraries were sequenced using an Illumina GAII system at the University of Virginia Biomolecular Research Facility. Integrative Genomics Viewer (IGV) was used to visualize the data. The scales were normalized to the Sir2 read count for each panel, based on the total number of mapped reads recovered.

### Standard ChIP assays

Standard ChIP assays to confirm the sequencing results were performed as previously described [26]. The preparation of chromatin solution from log-phase or post log-phase yeast culture is the same as described for the ChIP-seq procedure, except anti-myc antibody (9E10) and each specific antibody was combined with protein A/G conjugated sepharose beads blocked with salmon sperm DNA in the IP procedure. After incubation of chromatin solution with antibody and beads at 4°C overnight, the beads containing the immune-complex were washed (wash 1, twice with 1 ml of FA-140; wash 2, twice with 1 ml of FA-500 (the buffer was the same as FA-140 except that the NaCl concentration was increased to 500 mM); and wash 3, twice with 1 ml LiCl solution containing 10 mM Tris-HCl, pH 8.0, 250 mM LiCl, 0.5% NP-40, 0.5% sodium dodecyl sulfate, 1 mM EDTA). DNA was then eluted from the beads 2 times with 75 µl of elution buffer (5× TE plus 1% SDS). The combined DNA solution was incubated at 65°C overnight to reverse the cross-linking. The purified DNA samples were analyzed by quantitative real-time PCR, and the results normalized with the input DNA PCR signal, and indicated by relative IP in the graphs.

### Analysis of ChIP-sequencing reads

Sequence reads for Sir2, Sum1, Hst1, Hst2 libraries (ChIP DNA), and Input DNA were uniquely mapped to the SGD genome assembly (sacCer2) using BWA version 0.5.7 [83]. Reads that mapped uniquely were filtered on a phred quality score of 20, and were quantified as Sir2 (20760165), Hst1 (15530594), Sum1 (17613707), Hst2 (2637949), Hst3 (19078082), Hst4 (7210751), and Input (34090115). Repeats were allowed only on the rDNA locus on chromosome XII. Individual browsable enriched read wig files for Sir2, Sum1, Hst1, Hst2, and Input DNA were generated from the mapped read files by summing the number of overlapping reads for every genomic coordinate across the yeast genome.

Significant peaks were determined, relative to the Input DNA, using the BioConductor package, BayesPeak version 1.2.3 [43]. Subsequently, 1,391, 1,210, 1,527, and 146 peaks were highlighted as significantly enriched regions for Sir2, Sum1, Hst1, and Hst2 libraries respectively, after the posterior probability for each peak was required to exceed 0.5. Additional computational and statistical analyses are described in the Supplemental Information section in Additional file 1. The ChIP-seq datasets from this study have been deposited in NCBI's Gene Expression Omnibus, and are accessible through the GEO series accession number [GSE41415].

### Southern blotting

Genomic DNA (10 µg) was digested with *Xho*I at 37°C, and then separated on a 0.7% agarose gel, followed by transfer to an Immobilon-Ny^+ ^membrane (Millipore). The membrane was prehybridized in QuickHyb solution (Stratagene; Jolla, LA, USA) at 68°C for 20 minutes. To generate the telomeric repeat probe, a 350 bp *Eco*RI fragment was isolated from pYPLV [84], and labeled with [α^32^P] dCTP (3,000 Ci/mmol; Perkin Elmer; Boston, Massachusetts, USA) by random priming. The labeled probe was mixed with 100 µl of sonicated salmon sperm DNA (10 mg/ml), boiled to denature, and then hybridized to the membrane for 1 hour at 68°C in QuickHyb solution. The membrane was washed two times at room temperature in 2×-SSC + 0.1% SDS and once at 60°C in 0.1×-SSC + 0.1% SDS. The washed membrane was exposed to X-ray film for autoradiography.

### Quantitative reverse transcriptase (RT) PCR assays

Synthesis of cDNA from total RNA and PCR reactions with SYBR green PCR master mix was performed as previously described [26]. The oligonucleotide primer sequences are provided in Table S7 in Additional file 1. The test mRNA transcript levels were normalized to either *ACT1 *or *ALD2*. As indicated in some figures, to determine the fold induction, gene transcript levels in the mutant strains were also normalized to the levels in the wild-type strain. Results reflect the average fold induction (relative to the induction in the wild-type strain) from three biological replicates. Where indicated in the figures, the standard deviation was calculated.

### Glucose concentration measurements

The amount of glucose in the growth medium was assayed with a glucose assay kit (Sigma) using the glucose oxidase system. One milliliter of culture was removed at each time point from the indicated cultures and centrifuged at 10,000x g for 5 minutes to clarify the supernatant. The supernatant was further diluted 250-fold with deionized water and 250 µl of the diluted sample was subjected to the assay. Briefly, the reaction was started by adding 500 µl of assay reagent that contains o-dianisidine and glucose oxidase/peroxidase. After reacting for exactly 30 minutes at 37°C, the reaction was stopped by adding 500 µl of 12 N H_2_SO_4_, followed by measuring the absorbance at 540 nM.

### Intracellular NAD**^+ ^**and NADH measurements

To determine the NAD^+^/NADH ratio, we utilized a fluorescent NAD/NADH detection kit (Cell Technology, Inc; Mountain View, CA). Yeast cells were inoculated into 100 ml YPD medium and grown at 30°C in a shaker. A quantity of 2 × 10^6 ^cells was collected for each time point and then washed twice with 2 ml of phosphate-buffered saline. After removal of the final supernatant, the cell pellet was resuspended in 200 µl of the NAD or NADH extraction buffer supplied in the kit, and the rest of the protocol performed according to the manufacturer's instructions.

## Abbreviations

ChIP: chromatin immunoprecipitation; IGS: intergenic spacer; IGV: Integrative Genomics Viewer; IP: immunoprecipitation; ORF: open reading frame; rDNA: ribosomal DNA; TGM: tRNA gene-mediated; WT: wild type.

## Competing interests

The authors declare that they have no competing interests.

## Authors' contributions

ML contributed to conceptualization of the experiments, carried out most of the molecular biological experiments, and drafting of the manuscript. VV performed bioinformatic and statistical analysis with the ChIP-seq data and contributed to the writing. KP contributed to statistical analysis of the ChIP-seq data and diauxic shift gene expression analysis. SB contributed to conceptualization of the experiments, drafting the manuscript, and data analysis. JS conceived of the study, contributed to the experimental design, data analysis, and drafting of the manuscript. All authors read and approved the final manuscript.

## Supplementary Material

Additional file 1 **Supplementary online material**. This file contains Supplemental materials and methods, Figures S1 to S7, and Tables S1 to S7. Click here for file
