# Supplementary material for: Genome-wide analysis of functional sirtuin chromatin targets in yeast
Source: Genome Biol. 2013 May 27;14(5):R48. doi: 10.1186/gb-2013-14-5-r48 (PMC4053722; doi:10.1186/gb-2013-14-5-r48)
Supplement: Additional file 1 — Supplementary online material. This file contains Supplemental materials and methods, Figures S1 to S7, and Tables S1 to S7. [file gb-2013-14-5-r48-S1.PDF]

# Supplemental Information

## Materials and Methods

### Calculating the enrichment of genes bound by Sir2, Sum1 or Hst1 for differential regulation during the diauxic shift

Sir2, Sum1 and Hst1 bound ORFs (at least 60% covered) were compared to a set of genes that were previously identified as being differentially expressed during the diauxic shift using the Affymetrix Genechip platform [1]. The processed data was retrieved from dataset accession number E-TABM-496 at ArrayExpress. We used the differential $\log_2$ (fold-change) values to determine which genes were differentially expressed in the diauxic shift timepoint compared to log phase in WT strains. We then computed overlap of the Sir2, Sum1, or Hst1 bound genes with the differentially expressed gene list (either upregulated or downregulated). To calculate the p-value of association, we compared the overlap value with random overlaps of the dataset to account for enrichment or depletion over the random expectation (Figure S2b).

### Identification of condensin and cohesin sites

419 condensin binding sites were retrieved from the D'Ambrosio C et al, 2008 study [2]. PeakFinder [3] was used to determine 1248 cohesin peaks from Dataset S1 (W303 strain arrested by *cdc16-ts* with ChIP performed for Mcd1-18Myc), and mapped from sacCer1 to sacCer2 genomic coordinates using the LiftOver tool downloaded from the UCSC genome browser.

### Significance of overlaps between Sir2, Sum1 and Hst1 sites and condensin/cohesin sites, telomeric repeats and Pol III genes

Significance of overlaps of Sir2, Sum1 and Hst1 peaks with condensin and cohesin data (Table S5), telomeric repeats (Table S2), and Pol III transcribed genes (Table S4) was assessed using a random sampling model. All chromosomes were divided into mean peak size fragments. Thereafter, peaks were randomly sampled within each individual chromosome 10000 times. This approach preserved the total number and genomic coverage of peaks over the genome and each chromosome.

**Significance of overlaps of Sir2, Sum1 and Hst1 bound ORFs**

Significance of the high overlaps amongst Sir2, Sum1 and Hst1 bound ORFs (Figure 3C) was again assessed using a random sampling approach. Three different sets of lists were created by randomly sampling 105, 77 and 208 ORFs, corresponding to Sir2, Sum1 and Hst1 bound ORFs respectively, from all ORFs 10000 times. Mutual overlaps amongst the randomly selected ORFs were compared to the true overlaps to calculate the associated p-values and ratios of actual over mean random overlaps.

**Supplemental References**


1. Orzechowski Westholm J, Tronnersjo S, Nordberg N, Olsson I, Komorowski J, Ronne H: **Gis1 and Rph1 regulate glycerol and acetate metabolism in glucose depleted yeast cells.** *PLoS One* 2012, **7:**e31577.
2. D'Ambrosio C, Schmidt CK, Katou Y, Kelly G, Itoh T, Shirahige K, Uhlmann F: **Identification of cis-acting sites for condensin loading onto budding yeast chromosomes.** *Genes Dev* 2008, **22:**2215-2227.

3. Glynn EF, Megee PC, Yu HG, Mistrot C, Unal E, Koshland DE, DeRisi JL, Gerton JL: **Genome-wide mapping of the cohesin complex in the yeast Saccharomyces cerevisiae.** *PLoS Biol* 2004, **2:**E259.

## Supplemental Figure Legends

**Figure S1. ChIP-seq screenshots of Sirtuin and Sum1 binding to *HML*, *HMR*, and the rDNA genes.** The sequence read number scale for each row is shown in brackets. Each horizontal bar represents 500 bp.

**Figure S2. Confirmation ChIP and enrichment on genes downregulated during the diauxic shift. (a)** Confirmation ChIP assays of untagged, Hst2-myc, Sir2-myc, Hst1-myc, and Sum1-myc strains at the *ADH1* and *TDH3* genes. **(b)** Statistical analysis of Hst1, Sum1, or Sir2 enrichment on Pol II-transcribed genes that are differentially regulated during the diauxic shift. The box plot shows a trend toward association with downregulated genes (compared to all differentially regulated genes), and an apparent depletion for genes that are activated during the shift. The association with downregulated genes (green) was highly significant for Hst1. There was a significant depletion of each factor from the activated genes (red).

**Figure S3. Sir2, Hst1, Sum1, and Npt1 are required for ENO2 and CDC19 repression during the diauxic shift. (a)** RT-PCR analysis of *ENO2* expression relative to the *ACT1* control gene in WT, *sir2*Δ, *hst1*Δ, and *sum1*Δ strains growing exponentially. **(b)** RT-PCR analysis of *ENO2* expression during the diauxic shift in WT and the indicated mutant strains. **(c)** RT-PCR analysis of *ENO2* and *CDC19* expression during the diauxic shift in WT and *npt1*Δ strains.

**Figure S4. Sir2, Hst2, and Npt1 do not regulate the *CLB1*, *RIF1*, and *RPB3* genes during the diauxic shift. (a)** Screenshots of ChIP-seq reads across the ORF of each gene. No enrichment was observed compared to the Input control. **(b)** RT-PCR analysis of gene expression during the diauxic shift in WT, *sir2*Δ, *hst1*Δ, and *npt1*Δ strains.

**Figure S5. Quantitative ChIP assays of histone H4 and H3 acetylation levels in WT, *sir2*Δ *sum1*Δ, and *sir2*Δ *hst1*Δ double mutants.** The immunoprecipitated signal for each sample is relative to a mock IP sample with no antibody added, and then normalized to H3 or H4 occupancy. No significant increases in acetylation were observed in the mutants, compared to WT, for any of the acetylation marks tested.

**Figure S6. Venn diagram showing the number of Pol III-transcribed genes bound by Sir2, Hst1, or Sum1.**

**Figure S7. Condensin association with ribosomal protein genes. (a)** Western blot showing the steady state expression levels of Myc-tagged versions of Smc4, Scc2, and Mcd1 proteins in the various deletion mutants used throughout this study. α-tubulin was used as a loading control. **(b)** ChIP-assay showing the relative binding of Smc4-myc and Brn1-myc at the ribosomal protein genes *RPL10* and *RPL25*. The cells were grown to log phase in YPD medium.

Figure S1

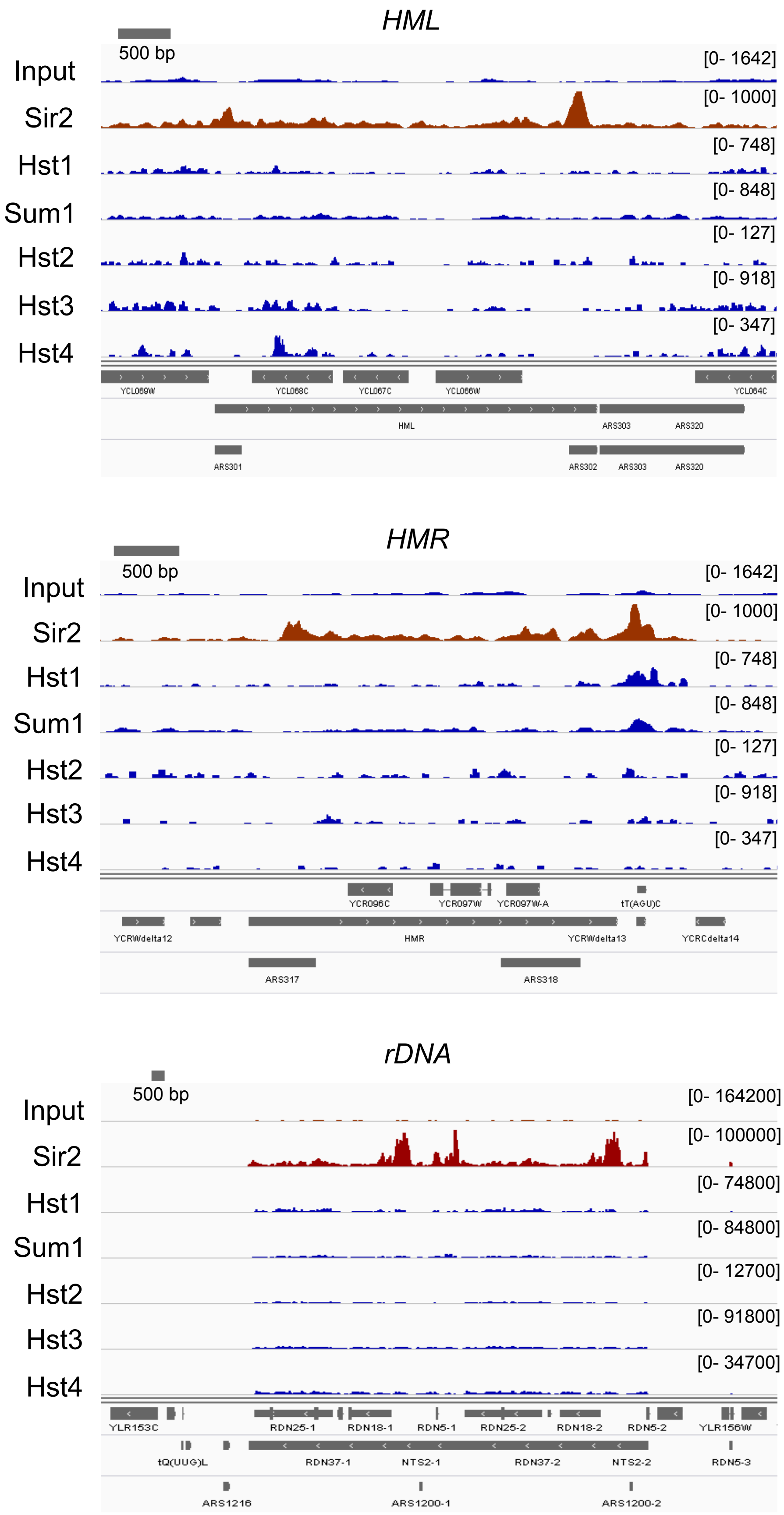
HML
500 bp
Input
Sir2
Hst1
Sum1
Hst2
Hst3
Hst4
[0- 1642]
[0- 1000]
[0- 748]
[0- 848]
[0- 127]
[0- 918]
[0- 347]
YCL069W
YCL068C
YCL067C
YCL066W
YCL064C
HML
ARS303
ARS320
ARS301
ARS302
ARS303
ARS320
HMR
500 bp
Input
Sir2
Hst1
Sum1
Hst2
Hst3
Hst4
[0- 1642]
[0- 1000]
[0- 748]
[0- 848]
[0- 127]
[0- 918]
[0- 347]
YCR096C
YCR097W
YCR097W-A
tT(AGU)C
YCRWdelta12
HMR
YCRWdelta13
YCRCdelta14
ARS317
ARS318
rDNA
500 bp
Input
Sir2
Hst1
Sum1
Hst2
Hst3
Hst4
[0- 164200]
[0- 100000]
[0- 74800]
[0- 84800]
[0- 12700]
[0- 91800]
[0- 34700]
YLR153C
RDN25-1
RDN18-1
RDN5-1
RDN25-2
RDN18-2
RDN5-2
YLR156W
tQ(UUG)L
RDN37-1
NTS2-1
RDN37-2
NTS2-2
RDN5-3
ARS1216
ARS1200-1
ARS1200-2

Figure S2

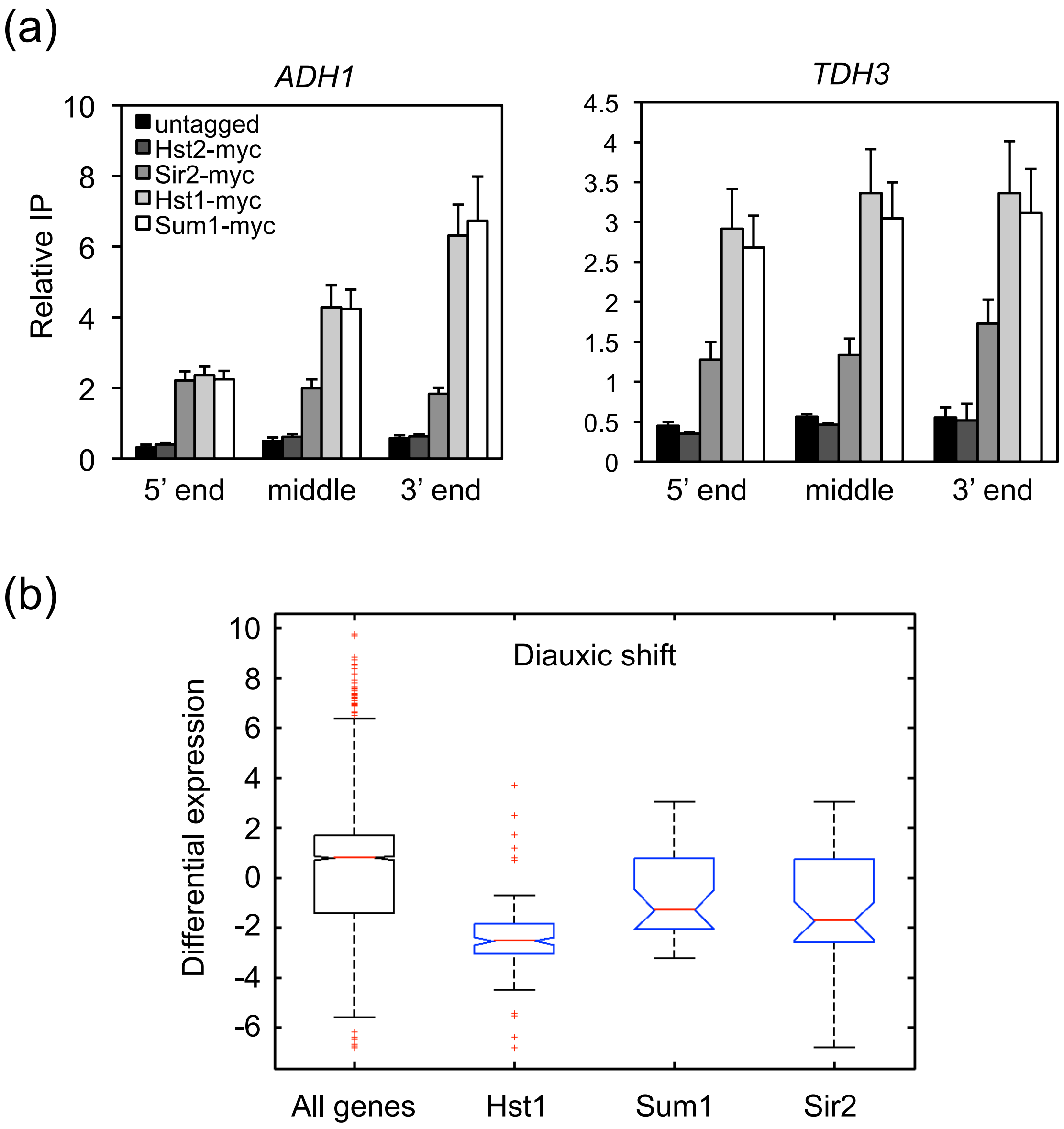


| | Hst1 | Sum1 | Sir2 |
|---|---|---|---|
| Genes with peaks | 208 | 77 | 105 |
| Repressed genes | 113 | 19 | 32 |
| p-values (enrichment) | **<0.0001** | 0.14 | 0.46 |
| Activated genes | 6 | 10 | 14 |
| p-values (depletion) | **<0.0001** | **<0.0001** | **<0.0001** |

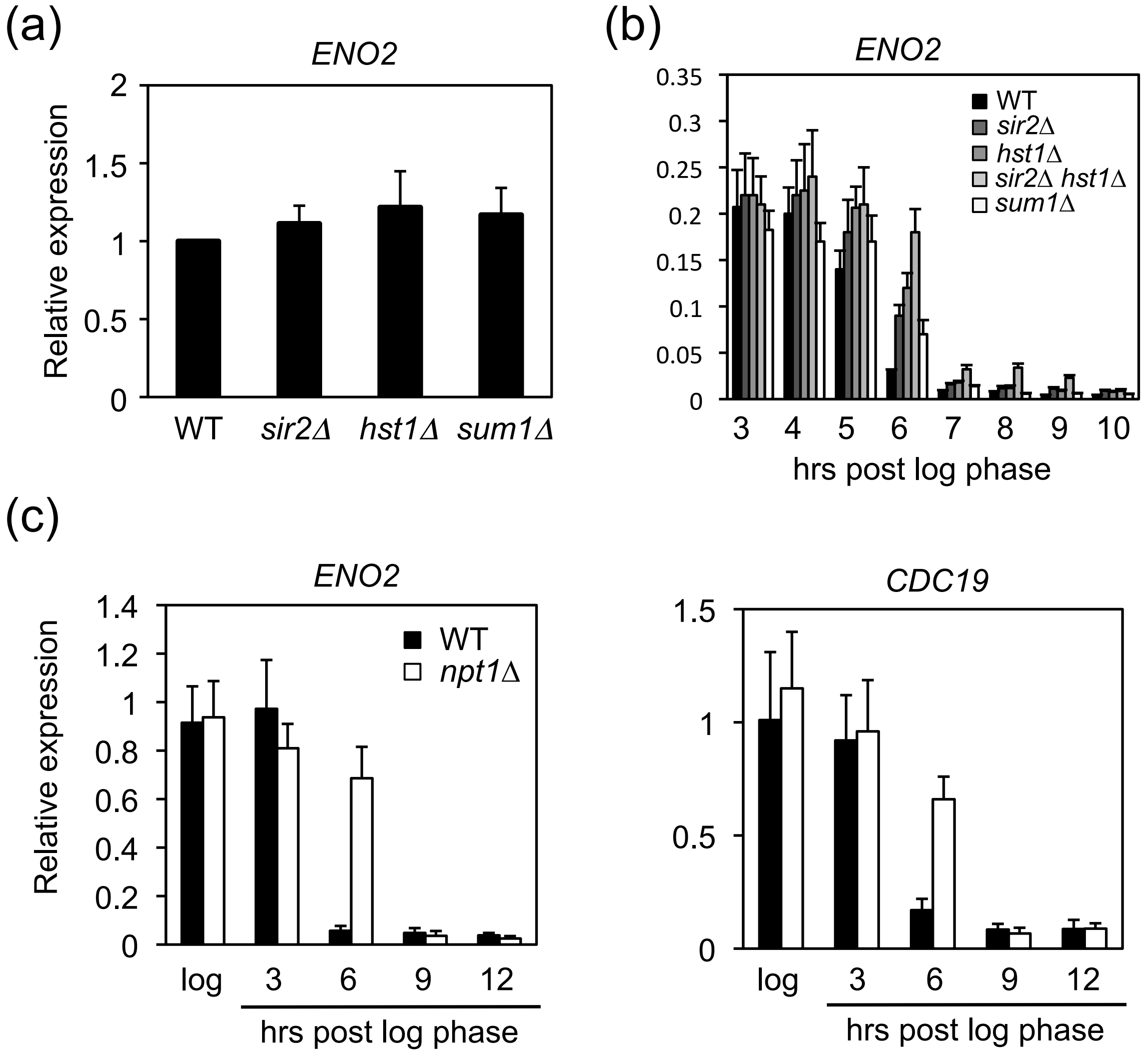
(a)
ENO2
Relative expression
2
1.5
1
0.5
0
WT
sir2Δ
hst1Δ
sum1Δ
(b)
ENO2
0.35
0.3
0.25
0.2
0.15
0.1
0.05
0
WT
sir2Δ
hst1Δ
sir2Δ hst1Δ
sum1Δ
3
4
5
6
7
8
9
10
hrs post log phase
(c)
ENO2
Relative expression
1.4
1.2
1
0.8
0.6
0.4
0.2
0
WT
npt1Δ
log
3
6
9
12
hrs post log phase
CDC19
1.5
1
0.5
0
log
3
6
9
12
hrs post log phase

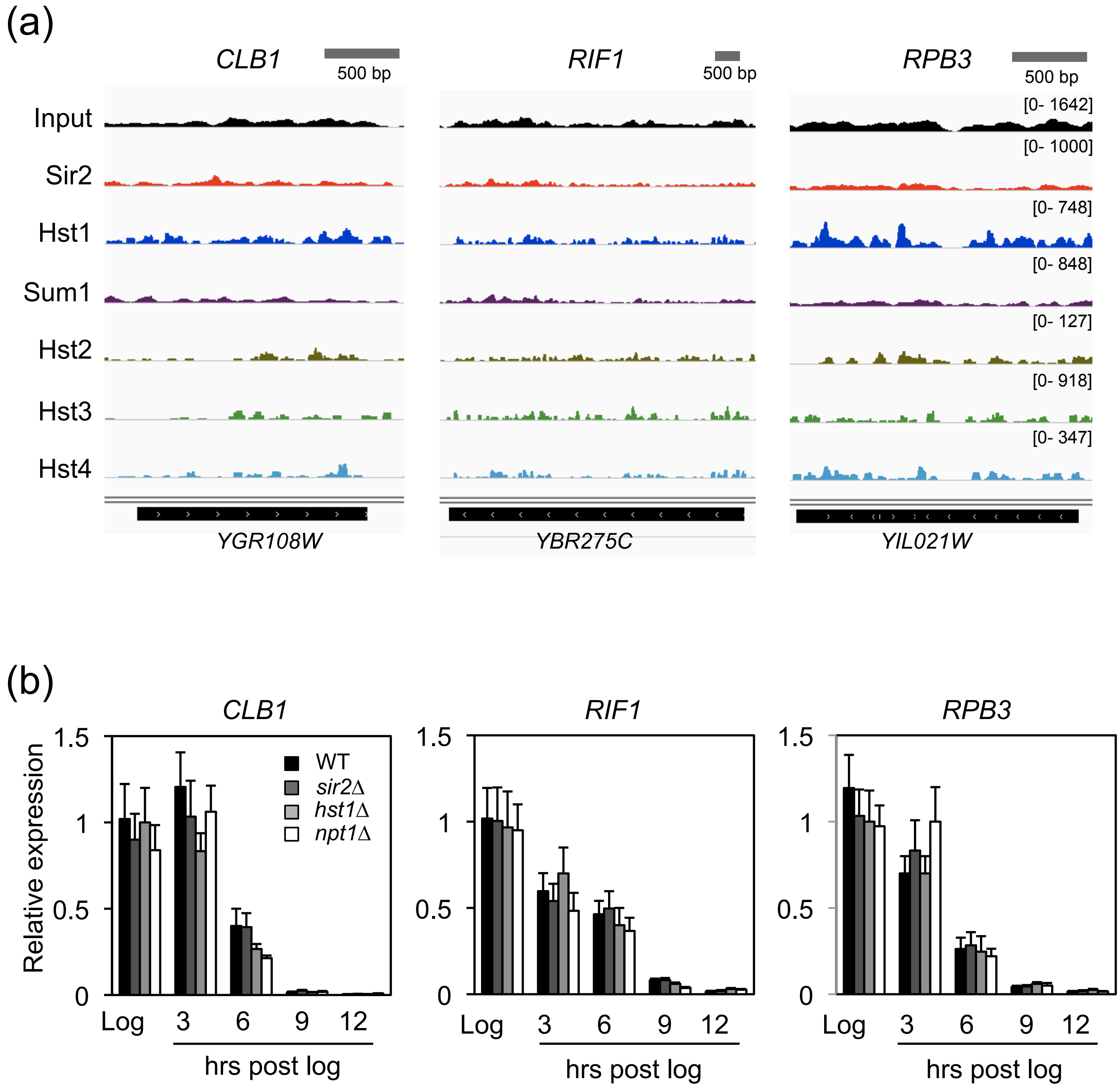
(a)
CLB1
500 bp
RIF1
500 bp
RPB3
500 bp
Input
Sir2
Hst1
Sum1
Hst2
Hst3
Hst4
[0- 1642]
[0- 1000]
[0- 748]
[0- 848]
[0- 127]
[0- 918]
[0- 347]
YGR108W
YBR275C
YIL021W
(b)
CLB1
RIF1
RPB3
Relative expression
1.5
1
0.5
0
WT
sir2Δ
hst1Δ
npt1Δ
Log
3
6
9
12
hrs post log

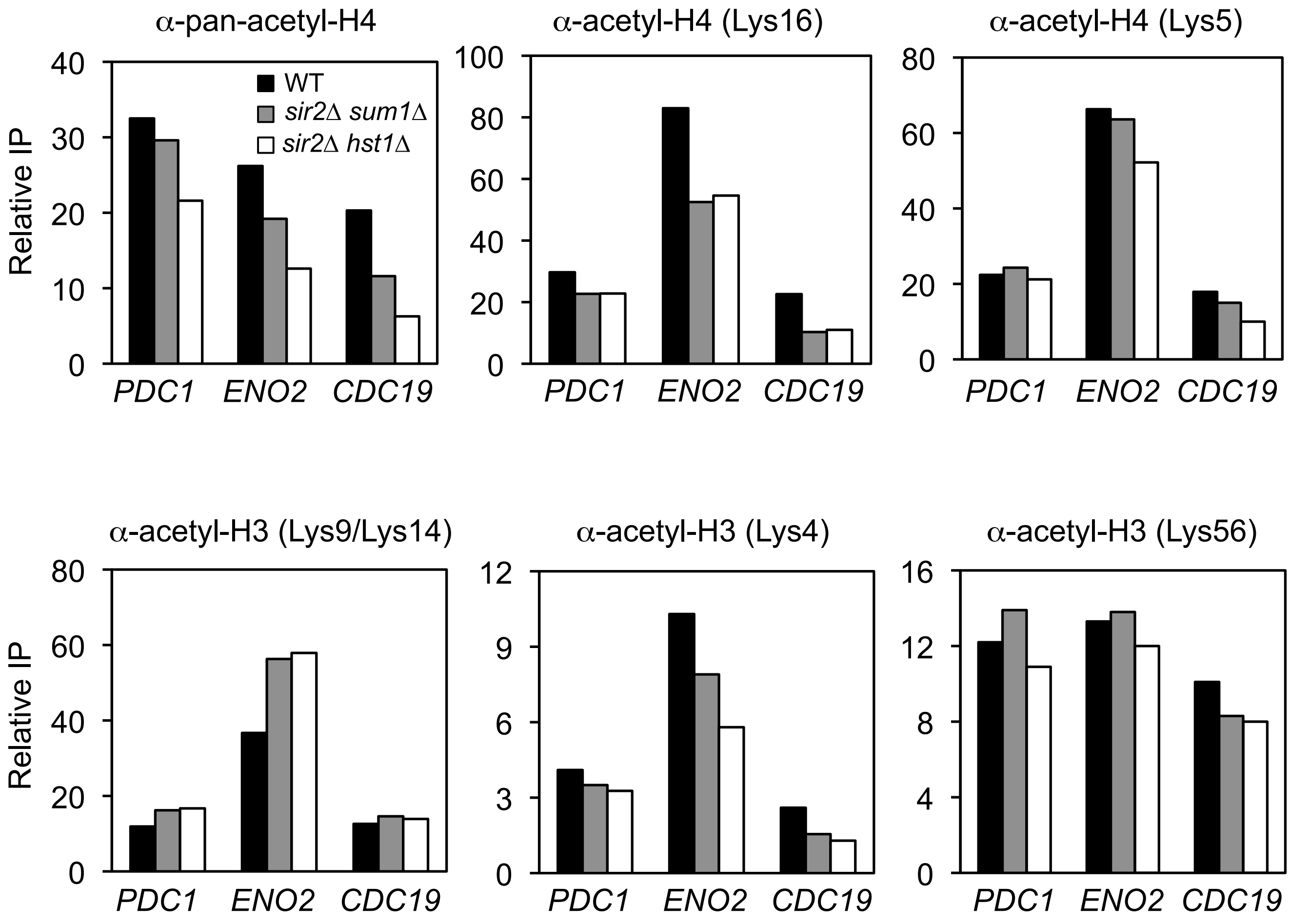
α-pan-acetyl-H4
α-acetyl-H4 (Lys16)
α-acetyl-H4 (Lys5)
α-acetyl-H3 (Lys9/Lys14)
α-acetyl-H3 (Lys4)
α-acetyl-H3 (Lys56)
Relative IP
WT
sir2Δ sum1Δ
sir2Δ hst1Δ
PDC1
ENO2
CDC19
0
10
20
30
40
60
80
100
3
6
9
12
4
8
16

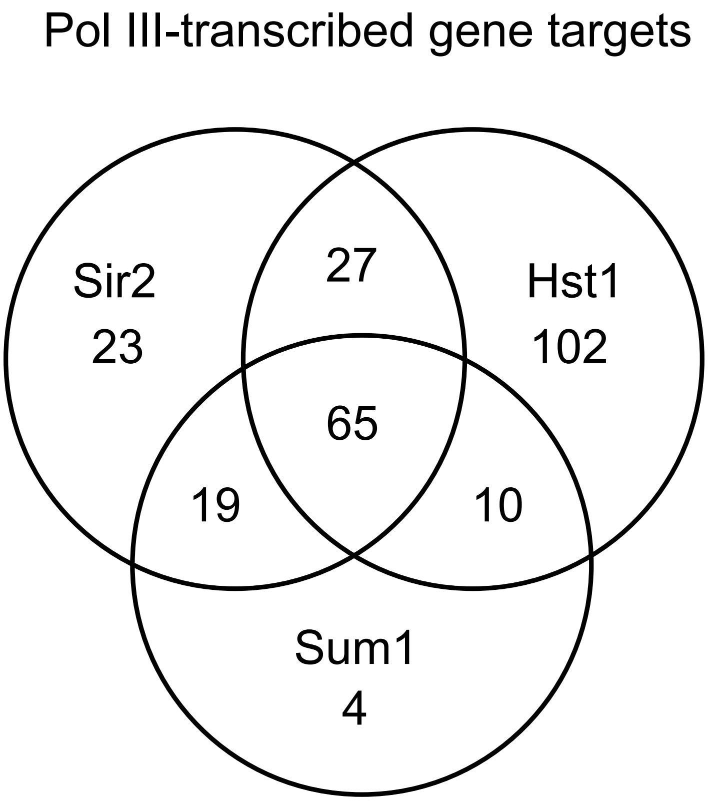
Pol III-transcribed gene targets
Sir2
23
27
Hst1
102
65
19
10
Sum1
4

(a)

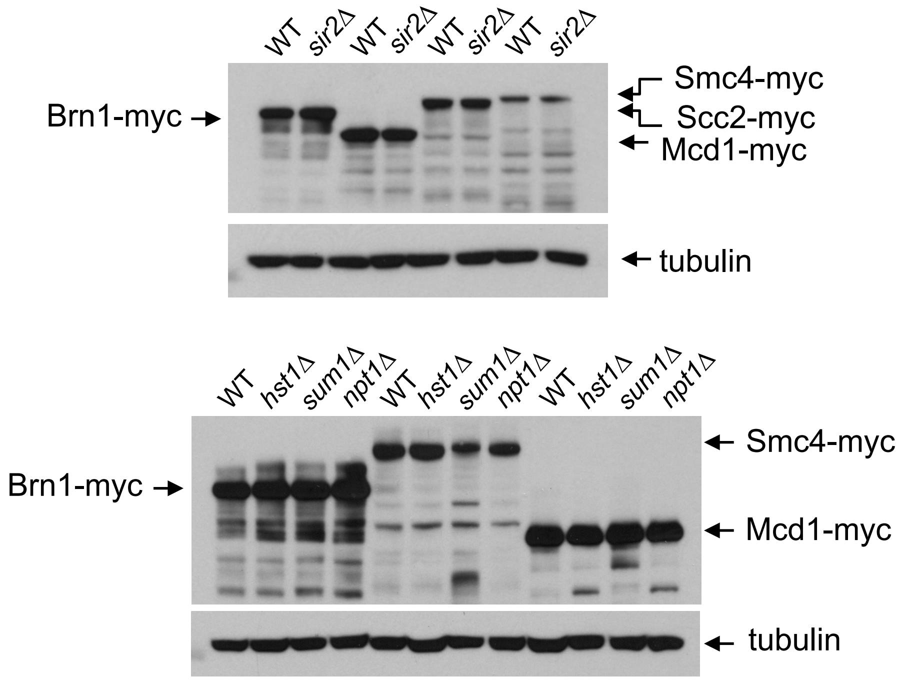
WT
sir2Δ
WT
sir2Δ
WT
sir2Δ
WT
sir2Δ
Brn1-myc
Smc4-myc
Scc2-myc
Mcd1-myc
tubulin
WT
hst1Δ
sum1Δ
npt1Δ
WT
hst1Δ
sum1Δ
npt1Δ
WT
hst1Δ
sum1Δ
npt1Δ
Brn1-myc
Smc4-myc
Mcd1-myc
tubulin

(b)

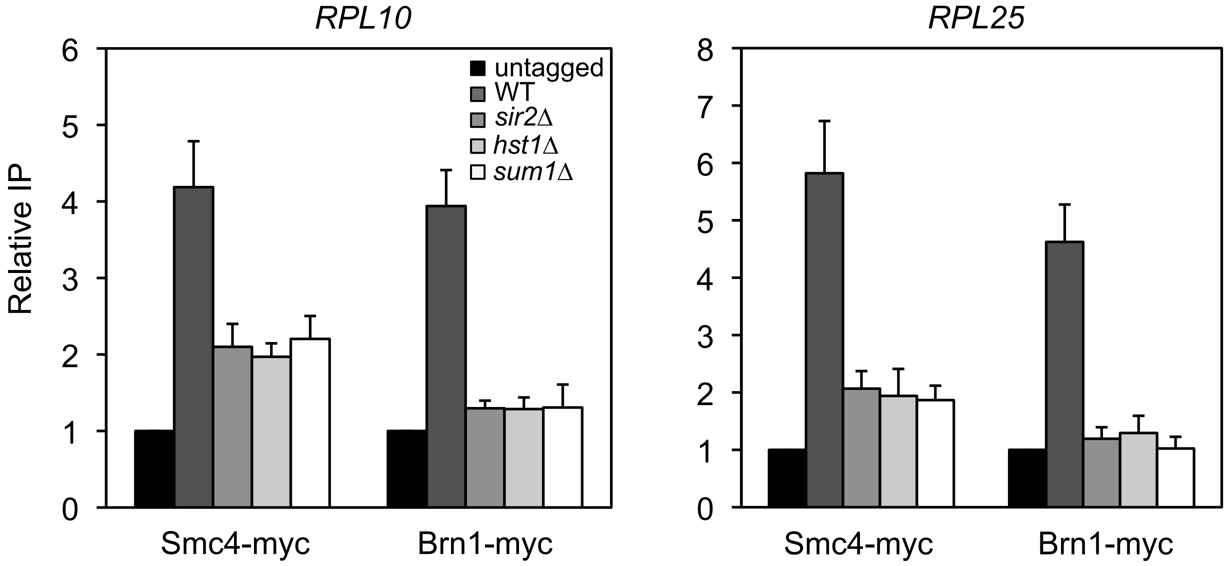
RPL10
RPL25
Relative IP
untagged
WT
sir2Δ
hst1Δ
sum1Δ
0
1
2
3
4
5
6
7
8
Smc4-myc
Brn1-myc
Smc4-myc
Brn1-myc

**Supplemental Table S1.** Terminal TG1-3 peak locations and intensities.

| | Sir2 | | Hst1 | | Sum1 | |
|---|---|---|---|---|---|---|
| Chr[1] | coordinate[2] | reads[3] | coordinate | reads | coordinate | reads |
| IL | 45 | 11492 | 45 | 3159 | 45 | 451 |
| IR | 230152 | 25441 | 230152 | 6848 | 230152 | 590 |
| IIR | 813147 | 13042 | 813147 | 4872 | 813147 | 665 |
| IIIL | 252 | 46874 | 252 | 14045 | 252 | 1506 |
| IIIR | 316564 | 20766 | 316564 | 6078 | 316564 | 710 |
| IVL | 117 | 25009 | 117 | 7795 | 119 | 705 |
| VIR | 270108 | 2549 | 270108 | 759 | 270107 | 120 |
| VIIIL | 52 | 14250 | 52 | 3853 | 52 | 1195 |
| VIIIR | 562525 | 35336 | 562525 | 14441 | 562525 | 1496 |
| IXL | 25-45 | 8098 | 25-45 | 2776 | 25-45 | 317 |
| IXR | 439841 | 6087 | 439841 | 1743 | 439835 | 245 |
| XL | 9-45 | 7341 | 9-45 | 2795 | 9-45 | 252 |
| XR | 745690 | 28255 | 745690 | 8953 | 745690 | 813 |
| XIL | 45 | 26123 | 45 | 9059 | 45 | 767 |
| XIR | 666290 | 65627 | 666290 | 23892 | 666290 | 2046 |
| XIIL | 80 | 30265 | 80 | 9210 | 80 | 3152 |
| XIIIL | 41-45 | 3037 | 41-45 | 1025 | 43 | 49 |
| XIIIR | 924334 | 16277 | 924334 | 6504 | 924334 | 458 |
| XIVR | 784202 | 39764 | 784201 | 14499 | 784201 | 1336 |
| XVL | 48 | 26828 | 48 | 9526 | 48 | 920 |
| XVR | ~1091268 | 6640 | ~1091268 | 1299 | ~1091268 | 395 |
| average | | 21862 | | 7292 | | 866 |
| Std. Dev. | | 15986 | | 5768 | | 734 |

[1]Chromosome termini with measureable $TG_{1-3}$ repeats

[2]SGD coordinate of the nucleotide(s) with the highest number of reads.

[3]Number of reads at the peak.

Table S2. Non-random enrichment of Sir2, Hst1 and Sum1 for telomeric repeats.

| | p value | No. of overlaps detected | Fold enrichment over random | Expected no. of overlaps (random) | SD random | z-score |
|---|---|---|---|---|---|---|
| Sir2 | < 1e-04 | 35 | 41.65 | 0.84 | 1.09 | 31.33 |
| Hst1 | < 1e-04 | 37 | 33.78 | 1.10 | 1.25 | 28.77 |
| Sum1 | < 1e-04 | 30 | 46.32 | 0.65 | 0.97 | 30.15 |

**Table S3.** GO Terms of Genes with Overlapping Sir2, Hst1, and Sum1 Binding to ORFs[1].

| GOBPID | Pvalue | OddsRatio | Expect | Count | Size | Term |
|---|---|---|---|---|---|---|
| GO:0006417 | 0 | 16.441 | 1 | 7 | 194 | regulation of translation |
| GO:0010608 | 0 | 15.106 | 1 | 7 | 210 | posttranscriptional regulation of gene expression |
| GO:0032268 | 0 | 14.586 | 1 | 7 | 217 | regulation of cellular protein metabolic process |
| GO:0051246 | 0 | 11.02 | 1 | 7 | 282 | regulation of protein metabolic process |
| GO:0006007 | 0 | 27.893 | 0 | 4 | 57 | glucose catabolic process |
| GO:0019320 | 0 | 25.033 | 0 | 4 | 63 | hexose catabolic process |
| GO:0046365 | 0 | 23.059 | 0 | 4 | 68 | monosaccharide catabolic process |
| GO:0046164 | 0 | 21.371 | 0 | 4 | 73 | alcohol catabolic process |
| GO:0019660 | 0 | 133.284 | 0 | 2 | 7 | glycolytic fermentation |
| GO:0019655 | 0 | 133.284 | 0 | 2 | 7 | glucose catabolic process to ethanol |
| GO:0044282 | 0 | 10.448 | 1 | 5 | 189 | small molecule catabolic process |
| GO:0006090 | 0 | 26.91 | 0 | 3 | 42 | pyruvate metabolic process |
| GO:0009063 | 0 | 24.976 | 0 | 3 | 45 | cellular amino acid catabolic process |
| GO:0044275 | 0 | 13.698 | 0 | 4 | 111 | cellular carbohydrate catabolic process |
| GO:0009310 | 0.001 | 22.79 | 0 | 3 | 49 | amine catabolic process |
| GO:0016052 | 0.001 | 12.728 | 0 | 4 | 119 | carbohydrate catabolic process |
| GO:0000955 | 0.001 | 74 | 0 | 2 | 11 | amino acid catabolic process via Ehrlich pathway |
| GO:0006006 | 0.001 | 12.617 | 0 | 4 | 120 | glucose metabolic process |
| GO:0006067 | 0.001 | 66.589 | 0 | 2 | 12 | ethanol metabolic process |
| GO:0034308 | 0.001 | 66.589 | 0 | 2 | 12 | monohydric alcohol metabolic process |
| GO:0046395 | 0.001 | 19.033 | 0 | 3 | 58 | carboxylic acid catabolic process |
| GO:0016054 | 0.001 | 19.033 | 0 | 3 | 58 | organic acid catabolic process |
| GO:0031323 | 0.001 | 4.598 | 3 | 10 | 1056 | regulation of cellular metabolic process |
| GO:0019318 | 0.001 | 10.974 | 0 | 4 | 137 | hexose metabolic process |
| GO:0010468 | 0.001 | 4.681 | 3 | 9 | 884 | regulation of gene expression |
| GO:0006113 | 0.001 | 47.534 | 0 | 2 | 16 | fermentation |
| GO:0010556 | 0.001 | 4.607 | 3 | 9 | 896 | regulation of macromolecule biosynthetic process |
| GO:0005996 | 0.001 | 9.838 | 1 | 4 | 152 | monosaccharide metabolic process |
| GO:0046165 | 0.001 | 15.833 | 0 | 3 | 69 | alcohol biosynthetic process |
| GO:0019752 | 0.001 | 6.032 | 1 | 6 | 400 | carboxylic acid metabolic process |
| GO:0043436 | 0.001 | 6.032 | 1 | 6 | 400 | oxoacid metabolic process |
| GO:0031326 | 0.001 | 4.507 | 3 | 9 | 913 | regulation of cellular biosynthetic process |
| GO:0006091 | 0.001 | 7.245 | 1 | 5 | 267 | generation of precursor metabolites and energy |

[1]Only GO terms with p-values 0.001 or lower.

**Table S4.** Statistically significant enrichment of Sir2, Hst1 and Sum1 for Pol III transcribed genes.

| | p value | No. of overlaps detected | Fold enrichment over random no. | Expected no. of overlaps (random) | SD random | z-score |
|---|---|---|---|---|---|---|
| Sir2 | < 1e-04 | 151 | 119.80 | 1.26 | 1.29 | 116.01 |
| Hst1 | < 1e-04 | 250 | 169.20 | 1.48 | 1.39 | 178.54 |
| Sum1 | < 1e-04 | 118 | 109.79 | 1.07 | 1.22 | 95.96 |

**Table S5.** Statistical analysis of condensin and cohesin overlap with Sir2, Hst1 and Sum1.

| | p value | No. of overlaps detected | Fold enrichment over random no. | Expected no. of overlaps (random) | SD random | z-score |
|---|---|---|---|---|---|---|
| *Condensin* | | | | | | |
| Sir2 | < 1e-04 | 97 | 173.28 | 0.56 | 0.76 | 126.49 |
| Hst1 | < 1e-04 | 170 | 208.82 | 0.81 | 0.92 | 184.33 |
| Sum1 | < 1e-04 | 65 | 139.49 | 0.47 | 0.70 | 91.86 |
| *Cohesin* | | | | | | |
| Sir2 | < 1e-04 | 40 | 28.85 | 1.39 | 1.23 | 31.28 |
| Hst1 | < 1e-04 | 47 | 24.07 | 1.95 | 1.46 | 30.90 |
| Sum1 | < 1e-04 | 34 | 28.94 | 1.17 | 1.15 | 28.58 |

**Table S6.** Yeast Strains.

| Strains | Genotype | Source |
|---|---|---|
| ML1 | *MAT*a *his3Δ200 leu2Δ1 met15Δ0 trp1Δ63 ura3-167* | [1] |
| ML2 | *MAT*a *his3Δ200 leu2Δ1 met15Δ0 trp1Δ63 ura3-167 npt1Δ::kanMX4* | [1] |
| ML28 | *MAT*α *his3Δ200 leu2Δ1 met15Δ0 trp1Δ63 ura3-167 sir2Δ::kanMX4* | [1] |
| ML29 | *MAT*α *his3Δ200 leu2Δ1 met15Δ0 trp1Δ63 ura3-167 hst1Δ::kanMX4* | [1] |
| ML30 | *MAT*α *his3Δ200 leu2Δ1 met15Δ0 trp1Δ63 ura3-167 sir2Δ::kanMX4 hst1Δ::kanMX4* | [1] |
| ML31 | *MAT*a *his3Δ200 leu2Δ1 met15Δ0 trp1Δ63 ura3-167 sum1Δ::kanMX4* | [1] |
| ML44 | *MAT*a *his3Δ200 leu2Δ1 met15Δ0 trp1Δ63 ura3-167 SIR2::13xMyc-kanMX4* | [1] |
| ML54 | *MAT*a *his3Δ200 leu2Δ1 met15Δ0 trp1Δ63 ura3-167 HST1::13xMyc-kanMX4* | [1] |
| ML65 | *MAT*a *his3Δ200 leu2Δ1 met15Δ0 trp1Δ63 ura3-167 SUM1::13xMyc-kanMX4* | [1] |
| ML96 | *MAT*a *his3Δ200 leu2Δ1 met15Δ0 trp1Δ63 ura3-167 HST2::13xMyc-kanMX4* | This study |
| ML149 | *MAT*a *his3Δ200 leu2Δ1 met15Δ0 trp1Δ63 ura3-167 BRN1::13xMyc-kanMX4* | This study |
| ML150 | *MAT*a *his3Δ200 leu2Δ1 met15Δ0 trp1Δ63 ura3-167 MCD1::13xMyc-kanMX4* | This study |
| ML152 | *MAT*a *his3Δ200 leu2Δ1 met15Δ0 trp1Δ63 ura3-167 SMC4::13xMyc-kanMX4* | This study |
| ML155 | *MAT*a *his3Δ200 leu2Δ1 met15Δ0 trp1Δ63 ura3-167 SMC4::13xMyc-kanMX4 hst1Δ::natMX4* | This study |
| ML157 | *MAT*a *his3Δ200 leu2Δ1 met15Δ0 trp1Δ63 ura3-167 BRN1::13xMyc-kanMX4 hst1Δ::natMX4* | This study |
| ML158 | *MAT*a *his3Δ200 leu2Δ1 met15Δ0 trp1Δ63 ura3-167 MCD1::13xMyc-kanMX4 hst1Δ::natMX4* | This study |
| ML160 | *MAT*a *his3Δ200 leu2Δ1 met15Δ0 trp1Δ63 ura3-167 SMC4::13xMyc-kanMX4 sir2Δ::natMX4* | This study |
| ML161 | *MAT*a *his3Δ200 leu2Δ1 met15Δ0 trp1Δ63 ura3-167 BRN1::13xMyc-kanMX4 sir2Δ::natMX4* | This study |
| ML163 | *MAT*a *his3Δ200 leu2Δ1 met15Δ0 trp1Δ63 ura3-167 MCD1::13xMyc-kanMX4 sir2Δ::natMX4* | This study |
| ML166 | *MAT*a *his3Δ200 leu2Δ1 met15Δ0 trp1Δ63 ura3-167 SMC4::13xMyc-kanMX4 sum1Δ::natMX4* | This study |
| ML168 | *MAT*a *his3Δ200 leu2Δ1 met15Δ0 trp1Δ63 ura3-167 MCD1::13xMyc-kanMX4 sum1Δ::natMX4* | This study |
| ML169 | *MAT*a *his3Δ200 leu2Δ1 met15Δ0 trp1Δ63 ura3-167* | This study |

| | | |
|---|---|---|
| | *BRN1::13xMyc-kanMX4 sum1Δ::natMX4* | |
| ML183 | *MAT*a *his3Δ200 leu2Δ1 met15Δ0 trp1Δ63 ura3-167 SMC4::13xMyc-kanMX4 npt1Δ::natMX4* | This study |
| ML184 | *MAT*a *his3Δ200 leu2Δ1 met15Δ0 trp1Δ63 ura3-167 MCD1::13xMyc-kanMX4 npt1Δ::natMX4* | This study |
| ML185 | *MAT*a *his3Δ200 leu2Δ1 met15Δ0 trp1Δ63 ura3-167 SCC2::13xMyc-kanMX4 npt1Δ::natMX4* | This study |
| ML187 | *MAT*a *his3Δ200 leu2Δ1 met15Δ0 trp1Δ63 ura3-167 SIR2::13xMyc-kanMX sir3Δ::natMX* | This study |
| ML188 | *MAT*a *his3Δ200 leu2Δ1 met15Δ0 trp1Δ63 ura3-167 SIR2::13xMyc-kanMX si4Δ::natMX* | This study |
| JS306 | *MAT*a *his3Δ200 leu2Δ1 met15Δ0 trp1Δ63 ura3-167 RDN1(18S)::mURA-HIS3 RDN1(NTS2)::Ty1-MET15* | [2] |
| JS1142 | *MATα his3Δ200 leu2Δ1 met15Δ0 trp1Δ63 ura3-167 RDN1 (NTS2)::Tyl-MET15* | This study |
| JS1143 | *MATα his3Δ200 leu2Δ1 met15Δ0 trp1Δ63 ura3-167 RDN1 (NTS2)::Tyl-MET15 sir2Δ::kanMX4* | This study |
| JS1144 | *MATα his3Δ200 leu2Δ1 met15Δ0 trp1Δ63 ura3-167 RDN1 (NTS2)::Tyl-MET15 hst1Δ::kanMX4* | This study |
| JS1145 | *MATα his3Δ200 leu2Δ1 met15Δ0 trp1Δ63 ura3-167 RDN1 (NTS2)::Tyl-MET15 sir2Δ::kanMX4 hst1::kanMX4* | This study |
| DSY364 | JS306 with *hst1Δ::kanMX4* | This study |
| DSY366 | JS306 with *hst2Δ::kanMX4* | This study |
| DSY368 | JS306 with *hst3Δ::kanMX4* | This study |
| DSY370 | JS306 with *hst4Δ::kanMX4* | This study |
| YG62 | *MATα RAP1 leu2-3,112 trp1 ade2-1 ura3-1 his3 adh4::ura3/ADE2-TELVIIL* | [3] |
| YG64 | *MAT***a** *rap1-17 leu2-3,112 trp1 ade2-1 ura3-1 his3 adh4::ura3/ADE2-TELVIIL* | [3] |
| SY675 | YG62 with *SIR2::13xMyc-kanMX4* | This study |
| SY676 | YG64 with *SIR2::13xMyc-kanMX4* | This study |
| SY677 | YG62 with *SUM1::13xMyc-kanMX4* | This study |
| SY679 | YG64 with *SUM1::13xMyc-kanMX4* | This study |
| SY680 | YG62 with *HST1::13xMyc-kanMX4* | This study |
| SY681 | YG64 with *HST1::13xMyc-kanMX4* | This study |

1. Li M, Petteys BJ, McClure JM, Valsakumar V, Bekiranov S, Frank EL, Smith JS: Thiamine biosynthesis in *Saccharomyces cerevisiae* is regulated by the $NAD^+$-dependent histone deacetylase Hst1. *Mol Cell Biol* 2010, 30:3329-3341.

2. Smith JS, Caputo E, Boeke JD: A genetic screen for ribosomal DNA silencing defects identifies multiple DNA replication and chromatin-modulating factors. *Mol Cell Biol* 1999, 19:3184-3197.

3. Obtained from David Shore. These strains were FOA-selected, and are $Ura^-$. They are derived from strains AJL275-2a (for YG62) and AJL440-1c (for YG64) from Arthur Lustig's lab.

**Table S7.** Oligonucleotides for qPCR.

| Target | DNA sequence |
|---|---|
| *PDC1* (ORF-5' end) sense | CTTCGGTGTCGGTGAATTGTC |
| *PDC1* (ORF-5' end) anti-sense | AGCTTGAGCAGAGATGGATGG |
| *PDC1* (ORF-middle) sense | CGATGCTGAATCCGAAAAGG |
| *PDC1* (ORF-middle) anti-sense | GACGTCGTGTCTGGAACAACA |
| *PDC1* (ORF-3' end) sense | CCATTGGTTTCACCACTGGTG |
| *PDC1* (ORF-3' end) anti-sense | TCAAGCCCCATCTGATCATG |
| *PDC1* (3'- intergenic) sense | GCCGACAGTCTGTTGAATTGG |
| *PDC1*(3- intergenic) anti-sense | GAAGCGGACCCAGACTTAAGC |
| *ENO2* (ORF-5' end) sense | TTTCGTCAAGGCCAACCTAGA |
| *ENO2* (ORF-5' end) anti-sense | CCAAGATAGCGTTAGCACCCA |
| *ENO2* (ORF-middle) sense | CTTGAACGTTTTGAACGGTGG |
| *ENO2* (ORF-middle) anti-sense | TCGGAACCAATTCTCATGGCT |
| *ENO2* (ORF-3' end) sense | CTAGAATTGCTACCG CCATCG |
| *ENO2* (ORF-3' end) anti-sense | GTTGGCAGCGAAAGAGTCTTG |
| *ADH1* (ORF-5' end) sense | TCACACTGACTTGCACGCTTG |
| *ADH1* (ORF-5' end) anti-sense | CCAGCCCTTAACGTTTTCACC |
| *ADH1* (ORF-middle) sense | AAGCCGCTCACATTCCTCAAG |
| *ADH1* (ORF-middle) anti-sense | ACCGGCCATCAAGTTAGCAGA |
| *ADH1* (ORF-3' end) sense | CAGCTGGTGCCAAGTGTTGTT |
| *ADH1* (ORF-3' end) anti-sense | AACCTCTGGCGAAGAAGTCCA |
| *TDH3* (ORF-5' end) sense | AACGGTTTCGGTAGAATCGGT |
| *TDH3* (ORF-5' end) anti-sense | AGCAGCGTAGTCGTTGGTGAT |
| *TDH3* (ORF-middle) sense | CCAACGCTTCTTGTACCACCA |
| *TDH3* (ORF-middle) anti-sense | ATCGTTGATAACCTTGGCCAA |
| *TDH3* (ORF-3' end) sense | AAGGCTGCCGCTGAAGGTA |
| *TDH3* (ORF-3' end) anti-sense | GCGTCTTCGGTGTAACCCAA |
| *CDC19* ORF sense | ACAACGCCAGAAAGTCCGAA |
| *CDC19* ORF anti-sense | CAATGGCCAATGGTCTACCTG |
| *PDC5* ORF sense | TCACAGTCGGCGCTCTATTG |
| *PDC5* ORF anti-sense | GATCAAGTTCTTCAGCGGCC |
| *CLB1* ORF sense | CGTCATTATGTGCCTCAGCG |
| *CLB1* ORF anti-sense | TGCCCAGCATTTTTCTCGA |
| *RIF1* ORF sense | AATTAGGCCAAGCCCACACAG |
| *RIF1* ORF anti-sense | CGTTACCATCTCCCCCCAA |
| *RPB3* ORF sense | GAAACTGACATGCGTTGCGA |
| *RPB3* ORF anti-sense | CACTTGGCGTGCTCTTTGG |
| *ALD2* ORF sense | CCAAGGTCGGTGGATTTGTG |
| *ALD2* ORF anti-sense | CTTTAAGGTTCGATTGGCCG |

| | |
|---|---|
| *BNA2* ORF sense | GTTGCTGACCTGCCCTCTCTT |
| *BNA2* ORF anti-sense | GACGCTCCGCACCTTGTTAT |
| tQ(UUG)H sense | ACTTTCGGTTTTGATCCGGA |
| tQ(UUG)H anti-sense | AAAGGTCCTACCCGGATTCG |
| tE(UUC)E1 sense | TGTAACGGCTATCACATCACGC |
| tE(UUC)E1 anti-sense | GGAGTCGAACCCCGGTCT |
| *SNR30* ORF sense | CAACGCTCGAGAGGCAGAG |
| *SNR30* ORF anti-sense | AATACGTGAGGCCCATTGGA |
| *RPL10* sense | GAAGAAGGCTACCGTCGATGA |
| *RPL10* anti-sense | TGGCACAGATACGAGCAGCTT |
| *RPL25* sense | TATTGACACTTTGGCAGCCG |
| *RPL25* anti-sense | TCAGCCAACTTGGAGACGAAT |
| *NTS1* sense | TGTTAGTGCAGGAAAGCGGG |
| *NTS1* anti-sense | CTACACCCTCGTTTAGTTGC |
| *NTS2* sense | GTATGTGGGACAGAATGTCG |
| *NTS2* anti-sense | GACTTACGTTTGCTACTCTC |
| ChrVI_TEL_70bp sense | GCCATTTCCGTGTGTAGTGA |
| ChrVI_TEL_70bp anti-sense | CCATGACCCAGTCCTCATTT |
| ChrVI_TEL_7000bp sense | TGCCACAATGCTATGGATTC |
| ChrVI_TEL_7000bp anti-sense | GTGAAAGTTCAGCGCAACAA |
